# Improved Recovery from Liver Fibrosis by Crenolanib

**DOI:** 10.3390/cells10040804

**Published:** 2021-04-04

**Authors:** Doreen Reichert, Louisa Adolph, Jan Philipp Köhler, Tobias Buschmann, Tom Luedde, Dieter Häussinger, Claus Kordes

**Affiliations:** Clinic of Gastroenterology, Hepatology and Infectious Diseases, Heinrich Heine University, Moorenstraße 5, 40225 Düsseldorf, Germany; doreen.reichert@hhu.de (D.R.); louisa.adolph@med.uni-muenchen.de (L.A.); janphilipp.koehler@med.uni-duesseldorf.de (J.P.K.); tobias.buschmann@hhu.de (T.B.); tom.luedde@med.uni-duesseldorf.de (T.L.); haeussin@uni-duesseldorf.de (D.H.)

**Keywords:** GATA4, HHEX, HNF4α, SOX17, liver fibrosis, stellate cells, cell stress, IRE1α, canonical WNT signaling

## Abstract

Chronic liver diseases are associated with excessive deposition of extracellular matrix proteins. This so-called fibrosis can progress to cirrhosis and impair vital functions of the liver. We examined whether the receptor tyrosine kinase (RTK) class III inhibitor Crenolanib affects the behavior of hepatic stellate cells (HSC) involved in fibrogenesis. Rats were treated with thioacetamide (TAA) for 18 weeks to trigger fibrosis. After TAA treatment, the animals received Crenolanib for two weeks, which significantly improved recovery from liver fibrosis. Because Crenolanib predominantly inhibits the RTK platelet-derived growth factor receptor-β, impaired HSC proliferation might be responsible for this beneficial effect. Interestingly, blocking of RTK signaling by Crenolanib not only hindered HSC proliferation but also triggered their specification into hepatic endoderm. Endodermal specification was mediated by p38 mitogen-activated kinase (p38 MAPK) and c-Jun-activated kinase (JNK) signaling; however, this process remained incomplete, and the HSC accumulated lipids. JNK activation was induced by stress response-associated inositol-requiring enzyme-1α (IRE1α) in response to Crenolanib treatment, whereas β-catenin-dependent WNT signaling was able to counteract this process. In conclusion, the Crenolanib-mediated inhibition of RTK impeded HSC proliferation and triggered stress responses, initiating developmental processes in HSC that might have contributed to improved recovery from liver fibrosis in TAA-treated rats.

## 1. Introduction

Our studies have demonstrated that hepatic stellate cells (HSC) are not only producers of extracellular matrix proteins but also exhibit the expression pattern and functional characteristics of mesenchymal stem cells (MSC), which becomes apparent when they are activated [1,2,3,4]. MSC form a heterogeneous group of somatic, multipotent stem cells that were first described in the bone marrow and are associated with blood vessels in all tissues [5,6]. MSC secrete trophic factors that support regenerative processes and can also differentiate into adipocytes, osteoblasts, chondrocytes, and myocytes, as well as liver epithelial cells, such as hepatocytes. Therefore, MSC have become an important research topic in the field of regenerative medicine [7,8,9,10]. However, beyond the potential beneficial effects of MSC various studies have demonstrated that MSC can also contribute to fibrosis in chronic diseases [11,12,13,14,15,16]. In the liver, HSC are associated with sinusoidal endothelial cells and located in the space of Disse, which constitutes their perivascular stem cell niche between endothelial cells and hepatocytes [4,17,18]. This niche preserves the quiescent state of HSC, which is characterized by the presence of retinoid-containing vesicles [19,20,21]. After liver injury, HSC become activated, lose their retinoids, and develop into myofibroblast-like cells, which contribute to fibrosis during chronic liver diseases through the deposition of extracellular matrix proteins [22] that affect vital liver functions.

The membrane-bound receptor tyrosine kinase (RTK) platelet-derived growth factor receptor (PDGFR) α and β are characteristic markers of MSC [3,23,24,25,26]. The binding of homo- or heterodimeric ligands comprised of PDGF-A, -B, -C, or -D protein chains with PDGFR initiates the phosphorylation of extracellular signal-regulated kinase (ERK)1/2 and phosphoinositide 3-kinase (PI3K)/protein kinase B (AKT), which results in enhanced cell proliferation and migration and reduced apoptosis rates [24,27,28]. Moreover, PDGFR signaling inhibit the differentiation of MSC into osteocytes, adipocytes, and myocytes [27,29,30], indicating that cell proliferation and differentiation represent opposing events. Because PDGFR signaling induces stellate cell proliferation [31,32], and the expression of PDGF and its receptors increase in patients with liver fibrosis [33,34,35,36], we examined whether the inhibition of PDGFR signaling by Crenolanib could influence HSC behaviors in the fibrotic liver. Crenolanib is an orally bioavailable benzamidazole derivate that can inhibit activated RTK, such as PDGFRα/β, FMS-related tyrosine kinase 3 (FLT3), and c-KIT, with the highest inhibitory effect for PDGFRβ [37,38]. In the present study, Crenolanib significantly improved liver recovery following thioacetamide (TAA)-induced fibrosis in rats. Crenolanib administration not only reduced PDGF-mediated proliferation but also initiated the hepatic endoderm specification of isolated HSC, which was dependent on p38 mitogen-activated protein kinase (MAPK) and c-Jun N-terminal kinase (JNK) activation. However, the developmental process remained incomplete in vitro, and the partially differentiated HSC accumulated lipids due to the activation of a stress response associated with inositol-requiring enzyme-1α (IRE1α).

## 2. Materials and Methods

### 2.1. Animal Model

**Figure 1:**
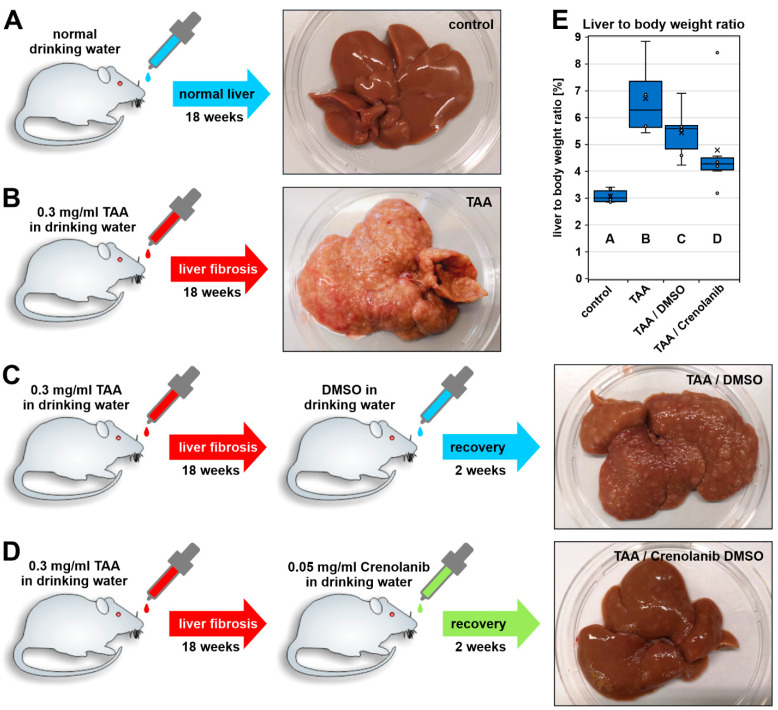
Experimental setup of the TAA-induced liver fibrosis model in rats. Rats were treated with 0.3 mg/mL thioacetamide (TAA) for 18 weeks via drinking water to induce liver fibrosis. (**A**) The control group received normal water and showed no changes in liver morphology, (**B**) the TAA-treated group exhibited the typical nodular liver surface characteristic of the fibrotic liver. (**C**,**D**) The livers were allowed to recover from liver fibrosis after the cessation of TAA treatment in the absence and presence of Crenolanib (0.05 mg/mL) for 14 days. (**C**) The control group with fibrosis was treated with an equal volume of the vehicle, dimethyl sulfoxide (DMSO), which was used as a solvent for Crenolanib. (**C**,**D**) Comparison of the liver morphology indicated accelerated liver regeneration in (**D**) the Crenolanib-treated group. (**E**) Improved recovery from fibrosis in the presence of Crenolanib was also indicated when the ratio of the liver to the body weight was determined (cross: arithmetic mean; line: median; boxes: 50% of the data; (**A**) *n* = 6; (**B**) *n* = 4; (**C**,**D**) *n* = 6).

Male Wistar rats (weight: 250–300 g; age: 8–10 weeks, *n* = 22) were obtained from Janvier Labs (Le Genest-Saint-Isle, France) and were treated with TAA (Sigma-Aldrich, Taufkirchen, Germany) via the drinking water for 18 weeks (Figure 1). TAA was initially administered at a concentration of 0.3 mg/L, which was then adapted according to the body weight of the rats [39]. The effects of TAA administration are well-known to induce liver fibrosis in Wistar rats [39,40,41]. After the cessation of TAA treatment, the Crenolanib-treated rats were allowed to recover from intoxication for 2 weeks in the presence of 0.05 mg/L Crenolanib (S2730; Selleckchem, Houston, TX, USA), which was dissolved in dimethyl sulfoxide (DMSO) and administered to the animals via the drinking water (*n* = 6). The Crenolanib concentration used here was adapted from experiments performed in mice (5 mg/kg body weight; [15]) and was adjusted according to the daily drinking water consumption of rats (10 mL/100 g). A daily uptake of 4.5 mg Crenolanib per kg body weight was recorded in our experiments. The control, without Crenolanib, received DMSO during the recovery period (TAA/DMSO; *n* = 6). Tissue samples were collected after perfusion of the liver with physiologic buffer at indicated time points. All animal experiments were approved by the local authority for animal protection (Landesamt für Natur, Umwelt und Verbraucherschutz Nordrhein-Westfalen, LANUV, Recklinghausen, Germany; reference number 81-02.04.2018.A126).

### 2.2. Cell Source, Culture, and Treatment

HSC were isolated from adult Wistar rats, which were obtained from the breeding colony of the Heinrich Heine University. The cell isolation protocol has been approved by the local authority for animal protection (LANUV, Recklinghausen, Germany; 82-02.04.2015.A287). The high lipid contents of HSC were used to enrich the cells through density gradient centrifugation (28% Nycodenz; Nycomed Pharma, Oslo, Norway) after the enzymatic digestion of the liver was performed as previously described [21]. Isolated HSC (1 million cells per 6-cm culture dish) were seeded in Dulbecco’s modified Eagle medium (DMEM; D6429; Sigma-Aldrich) supplemented with 10% fetal calf serum (FCS; Biochrom, Berlin, Germany) and 1% antibiotic/antimycotic solution (100×; Sigma-Aldrich). After one day, adherent HSC were washed with phosphate-buffered saline (PBS) and transferred to serum-free Iscove’s modified Dulbecco’s medium (IMDM; 12440-053; Gibco, Invitrogen, Karlsruhe, Germany) with 0.5 mg/mL bovine albumin (L9530, Sigma-Aldrich) in the presence of insulin (1% insulin-transferrin-selenite (ITS); I3146, Sigma-Aldrich) for 7 and 14 days. Clonally expanded HSC from rats (clone 5G4) were generated, as previously described [3], and cultured in a similar way. Serum-free medium was applied when the HSC clone reached confluency.

Crenolanib, the fibroblast growth factor (FGF) inhibitor BGJ398 (S2183; Selleckchem), the JNK inhibitor SP600125 (S1460; Selleckchem), the p38 MAPK inhibitor SB203580 (5633; Cell Signaling Technology, Danvers, MA, USA), the glycogen synthase kinase 3β (GSK3β) inhibitor TWS119 (S1590; Selleckchem), and the IRE1α inhibitor Kira6 (S8658; Selleckchem) were all dissolved in DMSO. For the short-term treatment of HSC (15–60 min), the inhibitors were applied for 1 h before Crenolanib was added. Cells in the control group were treated with the vehicle DMSO alone. To evaluate the inhibitory effects of Crenolanib on PDGFR signaling, HSC were pretreated with Crenolanib for 1 h before PDGF-BB (520-BB; R&D Systems, Minneapolis, MN, USA) was applied. In long-term experiments with Crenolanib (7–14 days), the inhibitors SP600125, SB203580, TWS119, and Kira6 were administered together with Crenolanib during the medium exchange. To identify mechanisms responsible for Crenolanib-mediated effects, HSC were treated with interleukin 1β (IL1β; 400-01B; PeproTech GmbH, Hamburg, Germany), lipopolysaccharide (LPS; L2630; Sigma-Aldrich), or dickkopf 1 (DKK1; abx066331; Abbexa Ltd., Cambridge, UK). All experimental media were changed completely every second to third day.

### 2.3. Cell Proliferation, Apoptosis, and Toxicity Assays

The proliferation of Crenolanib- and PDGF-BB-treated primary HSC cultures was determined under serum-free conditions by counting the living cells using a hemocytometer. The cells were treated with Crenolanib, PDGF-BB, or a combination of both substances for 72 h before being collected into a defined volume of physiologic buffer by trypsinization. The viability, cytotoxicity, and apoptosis rate of HSC were examined using the ApoTox-Glo Triplex Assay (Promega Corporation, Madison, WI, USA), according to the manufacturer’s recommendations. Freshly isolated HSC were seeded into 96-well plates at a density of 20,000 cells per well. After 2 days of culture, the cells were treated with 0.1 to 10 µM Crenolanib or vehicle control in a final volume of 100 µL per well. Additional control wells containing the appropriate medium without cells were also prepared to determine background fluorescence/luminescence. After 24 h of incubation, 20 µL viability/cytotoxicity reagent containing both GF-AFC substrate and bis-AAF-R110 substrate was added to all wells and briefly mixed by orbital shaking. The plates were incubated for 30 min at 37 °C, and the fluorescence was measured at 400 ex/505 em (viability) and 485 ex/520 em (cytotoxicity). A volume of 100 µL Caspase-Glo 3/7 reagent was added to all wells, briefly mixed by orbital shaking, and incubated for 30 min at room temperature. Finally, the luminescence was measured. For fluorescence and luminescence measurements, the GloMax-Multi+ Microplate Multimode Reader and the Instinct software (Promega Corporation) were used. Analysis was performed in quadruplicate.

### 2.4. Quantitative Polymerase-Chain Reaction (qPCR)

Total RNA was extracted from normal and injured rat livers and from isolated HSC using the RNeasy Mini Kit, according to the manufacturer’s instructions (Qiagen, Hilden, Germany). The RNA amount and quality were measured in a DropSense spectrometer (Trinean, Gentbrugge, Belgium). The Revert Aid H Minus First Strand cDNA Synthesis Kit (Thermo Fisher Scientific, Waltham, MA, USA) was used for cDNA synthesis. Quantitative polymerase-chain reaction (qPCR) was performed with the qTower3 cycler (Analytik Jena AG, Jena, Germany), and samples were measured in triplicate. For each PCR reaction, 25 ng cDNA and 0.6 µM of the appropriate primer pair (Supplemental Table S1) were combined with the Maxima SYBR Green qPCR Mastermix (Thermo Fisher Scientific) and subjected to a standard PCR program. Raw data were analyzed by the 2^(−ΔΔCt)^ method, normalized against the housekeeping genes hypoxanthine-guanine-phosphoribosyltransferase 1 (*Hprt1*) and ribosomal protein S6 (*Rps6*).

### 2.5. Immunoblotting

Protein analysis of whole-cell lysates was performed using the semidry Western blot technique, according to standard protocols. For each detection, 40 µg of whole protein lysates were separated using 10% sodium dodecyl sulfate-polyacrylamide gels and blotted on nitrocellulose membranes. The detection of protein bands was performed using primary antibodies, horseradish peroxidase (HRP)-coupled secondary antibodies (Supplemental Table S2), and Western Bright Quantum or Sirius HRP substrates (Advansta, Menlo Park, CA, USA). Visualization and documentation were performed with the ChemiDoc Imaging System (BioRad, München, Germany). Densitometric analyses of Western blot protein bands were performed using the multiplex band analysis tool of the AlphaView software (Proteinsimple, San Jose, CA, USA). The protein band intensities of phospho-proteins were normalized against the intensities of total ERK1/2, total AKT, total p38 MAPK, or total JNK protein, as appropriate. All other proteins were normalized against the intensity of γ-tubulin.

### 2.6. Immunofluorescence, Histochemistry, and Imaging

Cultured cells and liver tissue sections were fixed with ice-cold methanol (5 min) or 4% formalin (20 min). Formalin-fixed cells or sections were treated with 0.1% Triton-X-100 in PBS for 5 min. Unspecific antibody binding was prevented by blocking with 10% FCS for 1 h at room temperature. The primary antibodies (Supplemental Table S2) were diluted 1:100 in PBS containing 2% FCS and added to the cells and sections overnight at 4 °C. Secondary antibodies (Supplemental Table S2) were applied for 1–2 h in PBS containing 2% FCS at room temperature. The objects were finally mounted with Fluoromount G containing 4′,6-diamidino-2-phenylindole (DAPI, Southern Biotech, Birmingham, AL, USA) and a coverslip. To identify cellular lipid inclusions, HSC were washed with PBS and fixed with 4% formalin for 20 min. After the removal of the fixation solution, the cells were rinsed with distilled water and incubated in 60% isopropyl alcohol for 5 min. The isopropyl alcohol was aspirated, and the lipids within the cells were stained with aqueous 0.2% Oil-Red-O solution for 15 min. Finally, the Oil-Red-O was discarded, and the cells were washed several times with water. For the Sirius Red staining, liver sections were fixed with 4% formalin for 20 min, briefly washed in water, and treated with Sirius Red solution (0.2 g Sirius Red in 200 mL of 4% saturated picric acid (8 g picric acid in 200 mL distilled water); Sigma-Aldrich) for 60 min. The liver sections were washed twice in 0.01 N HCl for 2 min, dehydrated through an ethanol series, incubated in xylene, and finally mounted with Canada balsam (1.01691.0025; Merck, Darmstadt, Germany). Hematoxylin/eosin (HE) staining was performed after fixation of liver slides with ice-cold methanol for 5 min using hematoxylin solution (GILL II; GHS2; Sigma-Aldrich; diluted 1:5 in distilled water) for 5–10 min and eosin solution (eosin Y solution, alcoholic; HT1101; Sigma-Aldrich) for 2 min. After washing with ethanol and xylene, the sections were mounted with Canada balsam. Images were taken by a DP71 camera using the CellSens Dimension software (Olympus, Hamburg, Germany). The areas stained by Sirius Red or immunofluorescence on liver sections were determined using the FIJI imaging software [42].

### 2.7. CRISPR/Cas9

To knockout the gene interleukin-1 receptor-associated kinase 4 (*Irak*4) and inositol-requiring enzyme-1α (*Ire1*α), guide RNA (gRNA) for these genes were designed (Chopchop V3 [43]) for the target gene sequences CGGGGGCAACCGAATGGGAGAGG (Gene ID 300177) and CCGGGTCTTGGTGTCATACATGG (Gene ID 498013). The gRNA were synthesized with the GeneArt Precision gRNA Synthesis Kit (Invitrogen, Carlsbad, CA, USA), preincubated with the TrueCut Cas9 Protein v2 (Invitrogen), and finally transferred into freshly isolated HSC using the 4D-Nucleofector (program DS167; P3 Primary Cell 4D-Nucleofector X Kit L; Lonza, Basel, Switzerland). Control cells were treated identically, with the omission of gRNA. Transfected HSC were cultured for 3 days to determine the knockout efficiency and to stimulate the cells with 1 µM Crenolanib.

### 2.8. Statistics

At least 3 independent experiments were analyzed to calculate the mean and the standard error of the mean (±SEM), which was used to indicate data variation. Significant differences were determined by the non-parametric U- or H-tests, and *p*-values smaller than 0.05 were considered significant. Groups without significant differences share the same letters in graphs, whereas significantly different groups are indicated by diverse letters (a, b, c, d) or asterisks.

## 3. Results

### 3.1. Crenolanib Improves Recovery from Liver Fibrosis

**Figure 2:**
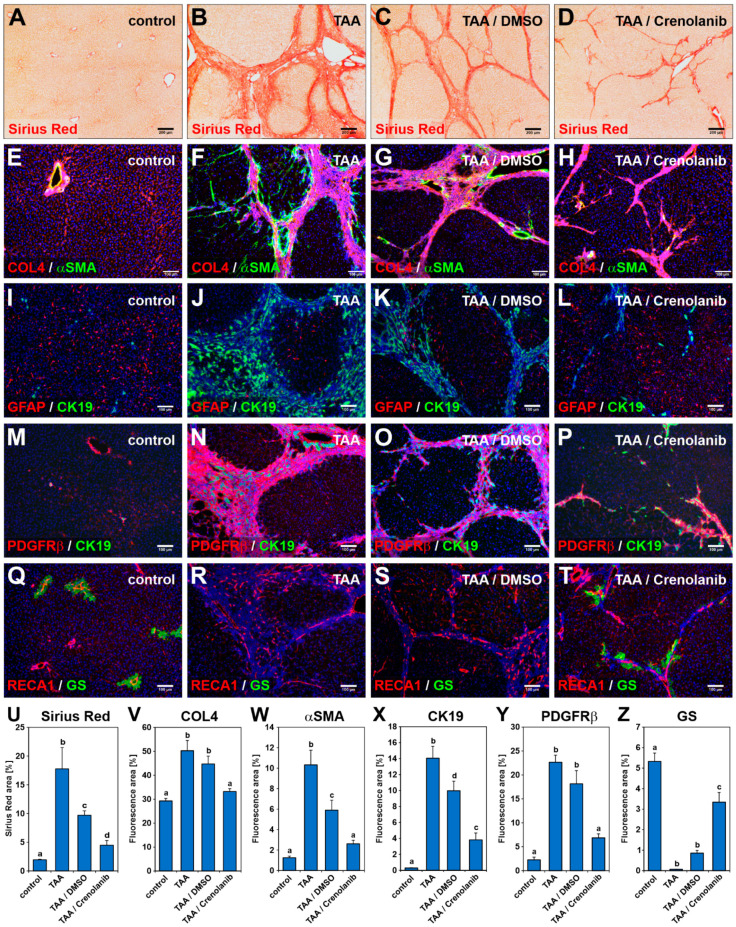
Crenolanib treatment improves liver fibrosis regression. Rats received (**A**,**E**,**I**,**M**,**Q**) normal (control) or (**B**,**F**,**J**,**N**,**R**) TAA-enriched drinking water for 18 weeks to induce liver fibrosis. After fibrosis induction, the rats were either treated with (**C**,**G**,**K**,**O**,**S**) DMSO or (**D**,**H**,**L**,**P**,**T**) Crenolanib (0.05 mg/mL) dissolved in DMSO for 14 days. Cryosections of liver samples obtained from these rats were subjected to (**A**–**D**) Sirius Red and (**E**–**T**) immunofluorescence staining, using antibodies against collagen 4 (COL4, red), α-smooth muscle actin (αSMA, green), glial fibrillary acidic protein (GFAP, red), platelet-derived growth factor receptor β (PDGFRβ, red), rat endothelial cell antigen-1 (RECA1, red), glutamine synthetase (GS) (green), and CK19 (green). (**D**,**H**,**L**,**P**,**T**) Rats from the Crenolanib-treated group showed weaker Sirius Red staining and decreased levels of COL4, αSMA, PDGFRβ, and CK19 staining. During fibrosis, hepatic stellate cells (HSC) activate and lower GFAP protein levels. The GS is expressed by a small subpopulation of hepatocytes around the central vein in normal liver, showing functional zonation of hepatocytes within the liver tissue. In the Crenolanib-treated group, recurrence of GFAP and GS staining was observed (**D**,**H**,**L**,**P**,**T**) compared with the DMSO control animals. (**T**) GS-expressing hepatocytes were always associated with RECA1-labeled endothelial cells and located near the residues of weakening scars. Cell nuclei were stained by DAPI (blue). Scale bars: 200 µm (**A**–**D**); 100 µm (**E**–**T**). (**U**–**Z**) The areas stained by Sirius red and immunofluorescence on liver sections (**A**–**T**) were quantified by computer-assisted methods, which confirmed the accelerated regeneration of the liver observed in the Crenolanib-treated group. The mean values of the control, TAA/Crenolanib, and TAA/DMSO groups represent data from 6 rats per group, whereas the TAA group represents the mean value of data from 4 rats (±SEM; *p* < 0.05; significant differences are indicated by different letters).

Rats were treated with TAA via drinking water for 18 weeks to initiate liver fibrosis, which was confirmed by the nodular appearance of the organ surface (Figure 1A,B). After the induction of fibrotic scars, the animals received either the vehicle DMSO or Crenolanib dissolved in DMSO via the drinking water for two additional weeks, at which time the livers were harvested (Figure 1C,D). When the livers were examined macroscopically, Crenolanib-treated rat livers showed a significant regression of nodules, and Crenolanib-treated rats had a reduced liver to body weight ratio compared with DMSO control rats (Figure 1D,E). The improved regeneration of fibrotic liver tissue in the presence of Crenolanib was also confirmed by microscopic analyses of tissue sections. Sirius Red staining (Figure 2A–D; Supplemental Figure SA1–D2), HE colorings (Supplemental Figure S1E–H), and the immunofluorescence intensity of collagen (COL) 4, alpha-smooth muscle actin (α-SMA), PDGFRβ, and cytokeratin (CK) 19 decreased in Crenolanib-treated rat livers compared with DMSO control rat livers (Figure 2E–P), suggesting the improved recovery from fibrosis. This recovery was also indicated by the increased abundance of glial fibrillary acidic protein (GFAP)-positive cells, which are characteristic of quiescent stellate cells, in the Crenolanib-treated group compared with the DMSO control group (Figure 2I–L).

Liver zonation was lost during liver fibrosis (Figure 2R) but was reestablished in the presence of Crenolanib, as indicated by the reappearance of glutamine synthetase (GS)-positive hepatocytes in proximity to blood vessels associated with scar residues (Figure 2Q–T). The quantitative assessment of Sirius Red-stained and immunofluorescent labeled (COL4, α-SMA, CK19, PDGFRβ, and GS) areas confirmed the occurrence of accelerated regeneration in the Crenolanib-treated group (Figure 2U–Z). In addition, the expression of hepatocyte-specific genes, such as *Gs*, hepatocyte nuclear factor 4α (*Hnf4α*), bile salt export pump (*Bsep*), and sodium/taurocholate co-transporting polypeptide (*Ntcp*) increased in whole liver tissue samples in the presence of Crenolanib compared with DMSO control animals, as investigated by qPCR (Supplemental Figure S1I–L).

### 3.2. Inhibition of RTK Signaling in Isolated HSC

**Figure 3:**
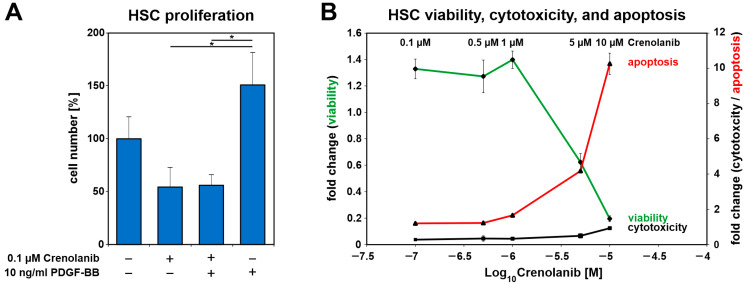
Crenolanib (≤1 µM) treatment inhibited PDGF-BB-mediated cell proliferation without adverse effects on HSC viability. (**A**) The proliferation of freshly isolated HSC in primary culture after Crenolanib (0.1 µM) and PDGF-BB (10 ng/mL) treatment was assessed after 72 h by cell counting under serum-free conditions (*n* = 3–4). PDGF-BB promoted the proliferation of HSC whereas Crenolanib inhibited PDGF-BB-mediated cell proliferation. (**B**) HSC survival was analyzed by the ApoTox-Glo assay after treatment with various concentrations of Crenolanib for 24 h (*n* = 4). Cell viability was assessed after the addition of the substrate GF-AFC, which is converted into a fluorescent dye by proteases, such as cathepsin C. The cytotoxicity of Crenolanib was determined using bis-AAF-R110, which is cleaved by proteases, such as tripeptidyl peptidase II, when the dye enters dying cells [44]. Apoptosis was measured in this experimental setup by determining caspase 3/7 activity. The data indicated that Crenolanib exerted no obvious adverse effects at the concentrations used in this study (0.1–1 µM). HSC survival was affected at concentrations of 5 and 10 µM Crenolanib.

**Figure 4:**
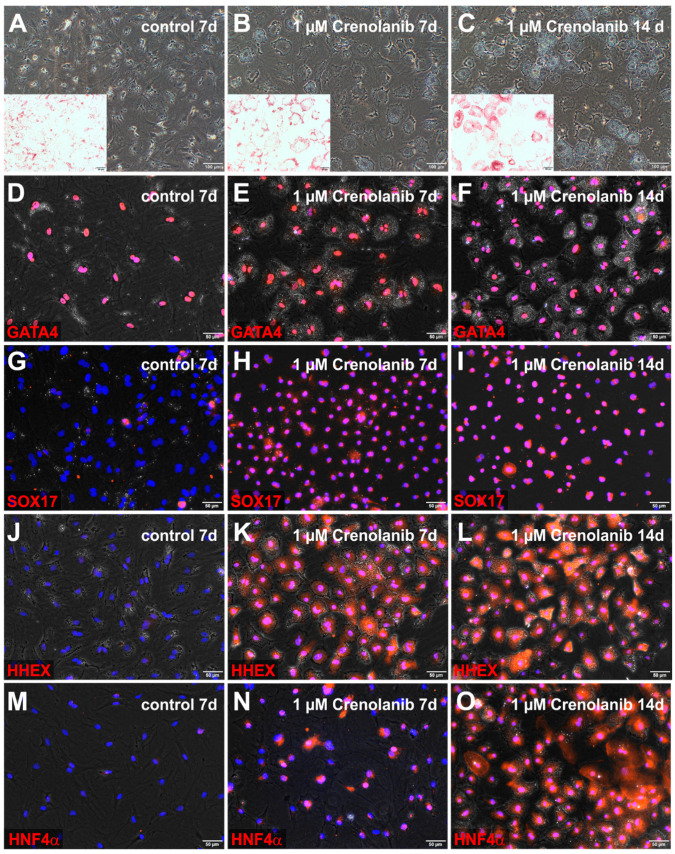
Crenolanib-induced endodermal markers in isolated rat HSC. HSC were cultured (**A**) without (control) and (**B**,**C**) with 1 µM Crenolanib for 7 to 14 days, using serum-free culture medium. In the presence of Crenolanib, the cells lost their myofibroblast-like cell morphology, became polygonal, and accumulated lipids, as determined by Oil-Red-O staining (red, inserts). (**D**–**O**) Immunofluorescence of GATA binding protein 4 (GATA4), SRY-box transcription factor 17 (SOX17), hematopoietically-expressed homeobox protein (HHEX), and hepatocyte nuclear factor 4α (HNF4α) in control and Crenolanib-treated HSC. (**D**–**F**) GATA4 (red) was always found in the cell nuclei of HSC, irrespective of treatment conditions. (**G**–**I**) SOX17 (red) was detectable in the cell nuclei of all HSC treated with Crenolanib, whereas SOX17 was occasionally stained in the cytoplasm of control HSC. (**J**–**L**) HHEX (red) appeared in the cell nuclei of all Crenolanib-treated HSC within the first week but was not detected in control HSC. GATA4 and HHEX remained detectable in Crenolanib-treated HSC after 14 days of treatment. (**M**–**O**) The endodermal transcription factor HNF4α (red) was found in the cell nuclei of (**N**) a subset of HSC within the first week of Crenolanib treatment, (**O**) but all HSC became positive for HNF4α after 14 days of Crenolanib exposure. Cell nuclei were stained by DAPI (blue). Scale bars: 100 µm (**A**–**C**); 50 µm (**A**–**C** inserts; **D**–**O**).

The RTK inhibitor Crenolanib preferably blocks PDGFRβ, and the administration of a low Crenolanib concentration (0.1 µM) significantly reduced PDGF-BB-mediated proliferation in HSC primary cultures under serum-free conditions compared with PDGF-BB treatment alone (Figure 3A). HSC survival, viability, and apoptosis were analyzed by the ApoTox-Glo assay after treatment with various concentrations of Crenolanib under serum-free conditions. Crenolanib exerted adverse effects at concentrations that exceeded 1 µM (Figure 3B). Even the prolonged treatment of HSC with 1 µM Crenolanib for seven days showed no obvious impairments of the cells, although the HSC acquired a polygonal cell shape and accumulated lipids during the extended culture time, as demonstrated by Oil-Red-O staining (Figure 4A–C).

The polygonal cell shape is a characteristic feature of hepatocytes, which prompted us to investigate whether Crenolanib-treated HSC were beginning to express liver progenitor cell and hepatocyte markers. The analysis of HSC treated with 0.1 µM Crenolanib for 7 days indicated that the expression of mesodermal markers that are known to be expressed by activated HSC such as desmin, collagen 1α2 chain (*Col1α2*), and *αSma* decreased compared their levels in untreated control cells as investigated by qPCR (Supplemental Figure S2A–C). Except for the fibrotic marker *Col1α2*, a comparable decrease was also observed for PDGF-BB-treated HSC (Supplemental Figure S2A–C). The liver progenitor cell markers *Ck19*, *Epcam*, and alpha-fetoprotein (*Afp*) and the hepatocyte markers cytokeratin 18 (*Ck18*), *Hnf1α*, *Hnf4α*, albumin, multidrug resistance-associated protein 2 (*Mrp2*), and forkhead box A2 (*Foxa2*) increased in HSC treated with Crenolanib. In contrast, the expression of *Afp*, *Hnf1α*, *Ck18*, and albumin decreased in response to PDGF-BB treatment (Supplemental Figure S2D–L). Although *Ck19* is also known to be expressed by cholangiocytes, other molecular markers of these bile duct cells, such as SRY-box transcription factor 9 (*Sox9*) and G protein-coupled bile acid receptor 1 (*Gpbar1/Tgr5*), remained undetectable by qPCR after Crenolanib treatment (data not shown), indicating that these cells were unlikely to be differentiating into cholangiocytes.

The induction of hepatocyte marker expression was also observed by qPCR in clonally expanded rat HSC (5G4 line) that were treated with increasing Crenolanib concentrations (0.1, 0.5, and 1 µM) for seven days. The HSC clone 5G4 was positive for PDGFRα and β, in addition to many proteins that are commonly used to characterize HSC and other MSC such as αSMA, SPARC like 1 (SPARCL1), nestin, vimentin, desmin, collagen 4, CD44, and CD146 (Supplemental Figure S3A–J). The expression of CD90 was low, and CK19 was absent in the HSC clone 5G4 (Supplemental Figure S3K,L). In agreement with primary rat HSC cultures, Crenolanib elevated the expression of liver progenitor cells (*Epcam*) and hepatocyte markers (*Hnf4α*, *Ck18*, albumin, bile salt export pump/*Bsep*) in a dose-dependent manner, based on the quantification of mRNA levels, which showed the strongest effect at 1 µM Crenolanib (Supplemental Figure S3M–Q).

### 3.3. Endodermal Specification of HSC

Crenolanib at the 1 µM concentration induced an epithelial cell morphology in all HSC within 7 days, which was maintained through at least 14 days (Figure 4A–C). The transcription factor GATA4, which is necessary for many developmental processes, including endoderm formation, was detectable in desmin-expressing HSC within liver sections and in freshly isolated HSC (Supplemental Figure S4A,B). GATA4 remained in the cell nuclei of HSC after Crenolanib treatment for up to 14 days (Figure 4D–F). The alteration of cell fate determination was indicated by the nuclear staining of SOX17 and HHEX in all HSC at days 7 and 14 of Crenolanib treatment (Figure 4G–L). By contrast, HNF4α first appeared in a subset of Crenolanib-treated HSC within 7 days, although all cells were positive for HNF4α after 14 days of stimulation (Figure 4M–O). GATA4, SOX17, HHEX, and HNF4α indicated endodermal specification of HSC. Although several hepatocyte-associated genes were induced in HSC by Crenolanib at the mRNA level, the corresponding protein synthesis remained low (e.g., FOXA2, CK18, and albumin; data not shown). This finding indicated that the continued exposure of HSC to Crenolanib could initiate hepatic endoderm specification but failed to support hepatic maturation.

### 3.4. Mechanism Underlying the Crenolanib-Mediated Endodermal Specification in HSC

**Figure 5:**
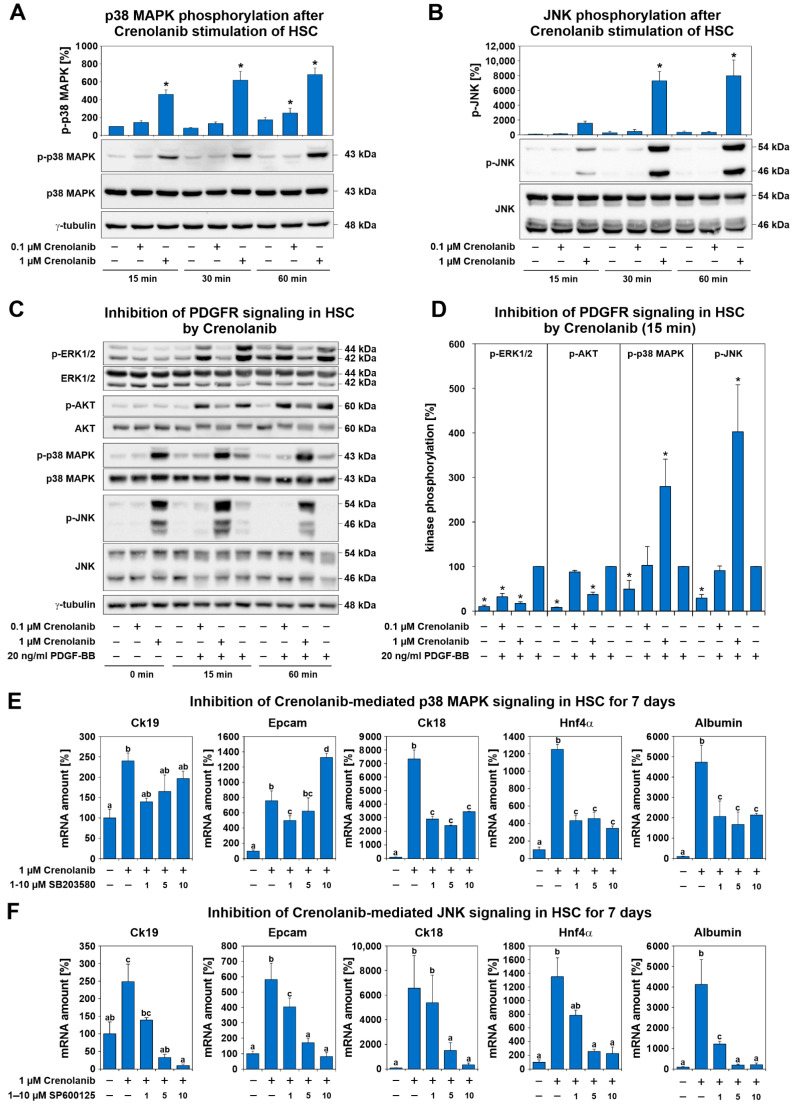
Crenolanib treatment enhanced p38 MAPK and c-Jun-activated kinase (JNK) activation in isolated rat HSC. (**A**,**B**) To identify the signaling pathways activated in HSC in the presence of Crenolanib, primary rat HSC were treated with this receptor tyrosine kinase (RTK) inhibitor (0.1 and 1 µM Crenolanib) and analyzed at the indicated time points by Western blot (*n* = 3). For the densitometry analysis of protein bands, total p38 MAPK or total JNK were used as the reference bands for p-p38 MAPK and p-JNK, respectively. Crenolanib induced the profound phosphorylation of p38 MAPK and JNK, especially at the 1 µM concentration. (**C**) The effects of Crenolanib on PDGFR signaling were investigated by the combined treatment of HSC that were pre-cultured for 7 days, followed by Western blot analysis (*n* = 3–6). (**D**) When the cells were pretreated with Crenolanib, the PDGF-BB-mediated phosphorylation of ERK1/2 and AKT was significantly inhibited compared with PDGF-BB treatment alone. PDGF-BB was set to 100% in the densitometry analysis of Western blots (*n* = 3–6). (**E**) Expression analysis of HSC treated with Crenolanib in the presence or absence of the p38 MAPK inhibitor SB203580. Increasing amounts of SB203580 were used, and the HSC were analyzed by qPCR after 7 days. The Crenolanib-mediated expression of liver progenitor cell markers *Ck19* and *Epcam* and the hepatocyte markers *Ck18*, *Hnf4α*, and albumin, were inhibited by SB203580. *Ck19* and *Epcam* expression were upregulated by high SB203580 concentrations. This inhibitor may interfere with other signaling pathways at higher concentrations (*n* = 4). (**F**) A similar approach was used to blocking JNK using SP600125, which resulted in liver progenitor cell and hepatocyte markers being efficiently suppressed in HSC (*n* = 4; * *p* < 0.05; significant differences are indicated by different letters).

To identify the molecular mechanism through which Crenolanib mediated the induction of hepatic endoderm in HSC, primary cultured rat HSC were treated with 0.1 or 1 µM Crenolanib for 15 to 60 min. Because p38 MAPK and JNK signaling have been reported to mediate cell differentiation processes [45,46,47], the phosphorylation of these kinases was investigated by Western blot analysis (Figure 5A,B).

Crenolanib elevated the phosphorylation level of p38 MAPK and JNK but significantly inhibited the PDGF-BB-mediated phosphorylation of ERK1/2 and AKT, as demonstrated by Western blot analysis (Figure 5C,D), which demonstrated the inhibitory effect of Crenolanib on PDGFR signaling. To elucidate whether the Crenolanib-induced hepatic differentiation of HSC was dependent on p38 MAPK and JNK phosphorylation, p38 MAPK (SB203580) and JNK (SP600125) signaling inhibitors were used. During short-term exposure experiments, 1 µM SB203580 significantly blocked the Crenolanib-induced phosphorylation of the MAPK-activated protein kinase 2 (MAPKAPK2) downstream of the p38 MAPK signaling cascade, whereas the phosphorylation of p38 MAPK was not significantly reduced (Supplemental Figure S5A). The JNK inhibitor SP600125 (5 µM) was able to reduce Crenolanib-mediated phosphorylation in HSC (Supplemental Figure S5B). The application of SB203580 (1, 5, and 10 µM) significantly reduced the expression levels of the hepatocyte-associated genes *Ck18*, *Hnf4α*, and albumin in HSC treated with 1 µM Crenolanib for 7 days, as determined by qPCR; however, p38 MAPK inhibition was unable to block the expression of hepatocyte-associated genes completely. The Crenolanib-induced expression levels of the liver progenitor cell markers *Ck19* and *Epcam* were also reduced, but this effect was lost at higher SB203580 concentrations (Figure 5E), which are known to exert side effects on other signaling pathways. By contrast, the JNK inhibitor SP600125 diminished the hepatic endoderm specification of HSC induced by 1 µM Crenolanib in a dose-dependent manner (Figure 5F). Thus, the p38 MAPK and JNK signaling pathways appeared to mediate the Crenolanib-induced developmental processes in HSC.

PDGF-BB exerted only weak effects on p38 MAPK and JNK phosphorylation, primarily stimulating ERK1/2 and AKT phosphorylation (Figure 5C,D). To elucidate the mechanism responsible for the pronounced phosphorylation of p38 MAPK and JNK, the expression of MAPK phosphatases (Mkp or dual-specificity phosphatase/Dusp) were analyzed during short- and long-term exposure experiments using 1 µM Crenolanib. Although the expression of some *Dusp* (*Dusp2*, *Dusp 7*, and *Dusp9*) genes decreased within 120 min after Crenolanib treatment (Supplemental Figure S6A–J), the application of Crenolanib for seven days, which was associated with sustained p38 MAPK and JNK phosphorylation in HSC was not followed by a significant reduction in *Dusp* expression at the mRNA level. Crenolanib either induced the upregulation of *Dusp* expression (e.g., *Dusp1*) or exerted no effect (Supplemental Figure S6K). However, the DUSP1 protein levels remained largely unchanged in HSC after either the short- or long-term exposure to Crenolanib, as determined by Western blot analysis (Supplemental Figure S7A,B).

Crenolanib also elevated the expression of hepatocyte growth factor (HGF) and fibroblast growth factors, such as *Fgf7* and *Fgf10*, which are known to support hepatic differentiation, but the increased expression of their corresponding receptors was only observed for *Fgfr3* at the concentration of 0.1 µM Crenolanib (Supplemental Figure S8A–D). The potential contribution of FGF signaling to the process of Crenolanib-mediated hepatic endoderm specification was examined through the application of the FGF inhibitor BGJ398 (0.2 µM), which did not counteract p38 MAPK and JNK phosphorylation (Supplemental Figure S8E–G).

IL1α/β and LPS signaling have been reported to activate p38 MAPK and JNK signaling [48,49], which was confirmed by Western blot analyses after stimulation of HSC with IL1β or LPS in the present study (Supplemental Figure S9A,B), suggesting that signaling via the interleukin 1 receptor (IL1R) and Toll-like receptor 4 (TLR4) could be responsible for p38 MAPK and JNK activation. Because IRAK4 is necessary for IL1R and TLR4 downstream signaling, the *Irak4* gene was deleted in HSC using clustered regularly interspaced short palindromic repeats (CRISPR)/CRISPR-associated (Cas9) technology. Although IRAK4 protein levels were efficiently reduced by up to 93% in HSC, the Crenolanib-mediated activation of p38 MAPK and JNK could not be prevented (Supplemental Figure S9C). Moreover, ERK1/2 was found to be activated in response to IL1β and LPS stimulation, which remained unaffected in Crenolanib-treated HSC (Supplemental Figure S9A,B), which suggested that IL1R and TLR4 signaling were not responsible for the Crenolanib-mediated effects observed in HSC.

**Figure 6:**
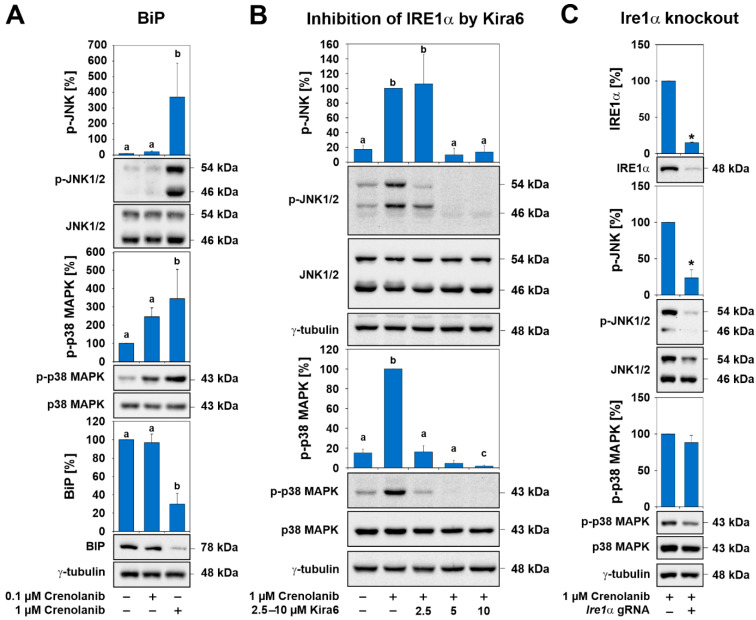
The effects of Crenolanib are mediated by a stress response via IRE1α. (**A**) The stimulation of culture-activated HSC with 0.1 and 1 µM Crenolanib for 7 days and the subsequent analysis of p-p38 MAPK, p-JNK, and binding immunoglobulin protein (BiP) by Western blot. Long-term exposure of HSC to 1 µM Crenolanib significantly lowered BiP levels by 70%. At this concentration, Crenolanib has also the greatest impact on JNK and p38 MAPK activation. (**B**) The inhibition of IRE1α by Kira6 in HSC significantly prevented the phosphorylation of p38 MAPK and JNK, as shown by Western blot analysis. (**C**) The CRISPR/Cas9 mediated knockout of *Ire1α* in HSC significantly reduced the Crenolanib-mediated phosphorylation of JNK but not p38 MAPK in comparison to the mock control, which was set to 100%. For densitometry, the protein bands for total p38 MAPK, total JNK, and γ-tubulin were used as references (*n* = 3; *p* < 0.05; significant differences are indicated by different letters or *asterisks).

Because lipids were found to accumulate in Crenolanib-treated HSC and lipid synthesis is known to be elevated during endoplasmic reticulum (ER) stress [50], the protein levels of binding immunoglobulin protein (BiP) were examined by Western blot analysis. A significant decrease in BiP levels was found in HSC exposed to 1 µM Crenolanib in long-term experiments for seven days (Figure 6A). BiP controls the activation of stress-associated IRE1α, which, in turn, activates p38 MAPK and JNK signaling [50]. To explore the contributions of IRE1α to the effects mediated by Crenolanib, IRE1α was inhibited by Kira6 before Crenolanib was applied. Both p38 MAPK and JNK phosphorylation were entirely impeded by Kira6 (Figure 6B). To verify this, *Ire1α* was deleted by CRISPR/Cas9 in HSC, and a knockout efficiency of 85–95% was achieved (Figure 6C). Although the p38 MAPK phosphorylation levels remained unchanged following Crenolanib administration to HSC with *Ire1α* knockout, JNK phosphorylation was significantly abolished (Figure 6C).

### 3.5. Regulatory Functions of Canonical WNT Signaling

**Figure 7:**
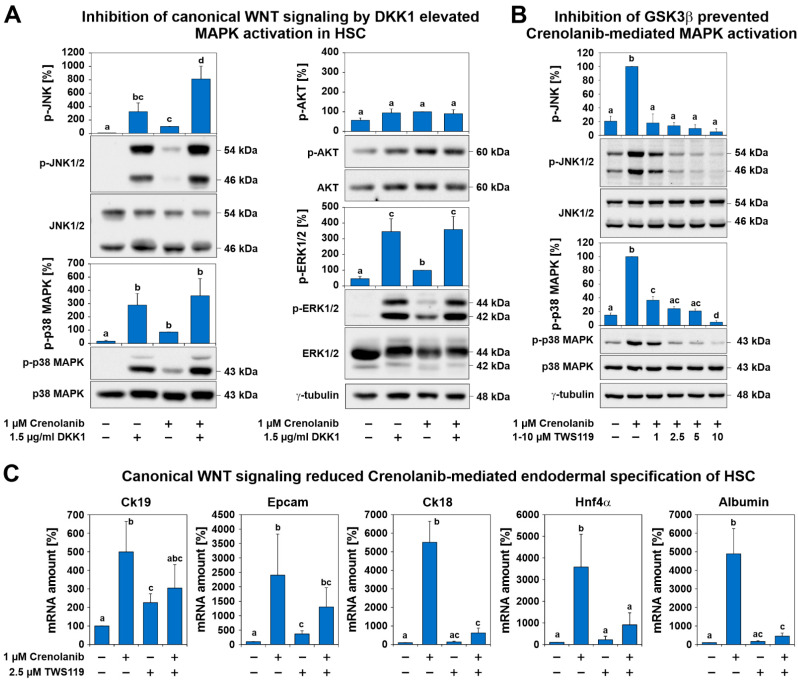
Canonical WNT signaling prevented the activation of signaling cascades associated with proliferation and differentiation, supporting quiescence in HSC. (**A**) Culture-activated HSC were treated with 1.5 µg/mL dickkopf 1(DDK1) for 1 h prior to 1 µM Crenolanib administration. The inhibition of canonical WNT signaling by DDK1 resulted in the increased phosphorylation of p38 MAPK, JNK, and ERK1/2, as assessed by Western blot analysis, even in the absence of Crenolanib, whereas AKT signaling was weakly affected. Crenolanib significantly elevated the phosphorylation of JNK (*n* = 3; *p* < 0.05). (**B**) HSC were pretreated with the GSK3β inhibitor TWS119, which reduces β-catenin degradation and promotes canonical WNT signaling. This approach significantly reduced the activation of p38 MAPK and JNK in response to Crenolanib. The results of the Crenolanib-treated group were set to 100% in the densitometry analysis of the Western blots (*n* = 3). (**C**) The expression analysis of HSC treated with Crenolanib for 7 days in the presence or absence of 2.5 µM TWS119. The Crenolanib-induced expression of hepatocyte markers *Ck18*, *Hnf4α*, and albumin was inhibited by TWS119, as measured by qPCR. The progenitor markers *Epcam* and *Ck19* were only slightly suppressed (*n* = 4; *p* < 0.05; significant differences are indicated by different letters).

The inhibition of β-catenin-dependent or canonical WNT signaling by DKK1 via LRP5/6 inhibition enhanced the activation of p38 MAPK, JNK, and ERK1/2. DKK1 was able to mediate this process even in the absence of Crenolanib (Figure 7A). HSC were pretreated with the GSK3β inhibitor TWS119, which reduces β-catenin degradation and promotes canonical WNT signaling. This approach efficiently reduced p38 MAPK and JNK activation and endodermal specification in response to Crenolanib (Figure 7B,C). Thus, canonical WNT signaling prevented the activation of signaling cascades associated with proliferation and differentiation, supporting quiescence in HSC (Figure 7).

## 4. Discussion

Crenolanib is an inhibitor of RTK signaling that is currently being tested in clinical trials for the therapy of various tumors (https://clinicaltrials.gov/, accessed on 4 April 2021). In the present study, we observed that Crenolanib improved the recovery from liver fibrosis in rats treated with TAA. The TAA model used in our present study simulates the situation in which patients with preexisting liver fibrosis seek medical treatment and in which the etiology of chronic liver disease is known and treatable. In this scenario, Crenolanib represents a promising therapeutic option that might enhance the regression of liver fibrosis in patients. RTK signaling, such as the PDGFR-mediated pathways, is well-known to induce cell proliferation in HSC [31], contributing to the formation of fibrous scars. Interestingly, the inhibition of RTK signaling in HSC by Crenolanib not only reduced PDGF-mediated cell proliferation but also reduced the expression of mesodermal markers and induced the expression of endodermal transcription factors, such as SOX17, HHEX, and HNF4α, which became detectable in all Crenolanib-treated HSC over time. SOX17 and HHEX are required for gut endoderm formation and early liver development, as shown in knockout mouse models [51,52,53]. In addition, nuclear HNF4α denotes hepatoblasts during embryonal liver bud formation, and the transcription factor GATA4 is involved in early liver development [54]. GATA4 is also expressed by hepatocytes, in addition to HSC in rats, and declining GATA4 levels have been associated with liver fibrosis [55,56]. Although the function of GATA4 in HSC is not currently known, GATA4, SOX17, HHEX, and HNF4α can be regarded to serve as key transcription factors for endodermal fate specification. In addition, Crenolanib-treated-HSC developed polygonal cell morphology, which is characteristic of hepatocytes; however, the further maturation into hepatocytes could not be observed in vitro, and many hepatocyte-associated molecular markers remained weak at the protein level. Instead, lipids began to accumulate in HSC in response to Crenolanib treatment.

To unravel the molecular mechanisms responsible for the Crenolanib-mediated initiation of endodermal specification in HSC, the signaling pathways that are known to regulate cell proliferation and differentiation were analyzed. Although PDGF-BB-mediated ERK1/2 phosphorylation was significantly suppressed by Crenolanib, the p38 MAPK and JNK signaling pathways were markedly elevated and found to be responsible for the Crenolanib-mediated endodermal specification of HSC. Thus, after blocking the RTK with Crenolanib, those pathways capable of triggering p38 MAPK and JNK phosphorylation appear to dominate the signaling in HSC leading to the initiation of cell development. This finding is in line with previous observations by other groups, which demonstrated the involvement of p38 MAPK and JNK signaling during the cell differentiation processes [45,46,47]. In vitro, Crenolanib has been shown to exert long-lasting effects that persist for at least two days on the activation state of MAPK, as observed in the present study.

In the search for the pathways responsible for p38 MAPK and JNK activation in the absence of RTK signaling, FGFR-, TLR-, and IL1R-mediated signaling pathways were investigated. Blocking FGF signaling and IRAK4, which is a downstream element of ILR and TLR signaling [57], using either small inhibitory molecules or CRISPR/Cas9 could not prevent Crenolanib-mediated p38 MAPK and JNK activation. Moreover, MAPK phosphatases, which might contribute to sustained kinase activation in response to Crenolanib, showed no increased expression during long-term experiments. However, environmental stressors trigger the adaptive responses of cells via p38 MAPK and JNK activation, which are mechanisms that have also been associated with altered lipid metabolism and obesity [50,58]. The activation of the stress response could explain the observed Crenolanib-mediated lipid accumulation and the imperfect developmental processes in HSC. In line with this, the ER chaperone BiP decreased in HSCs treated with 1 µM Crenolanib for two days, which may indicate the activation of the ER stress sensors IRE1α, protein kinase R (PKR)-like ER kinase (PERK), and activating transcription factor 6 (ATF6), which are typically prevented from dimerization and activation by BiP in unstressed cells. During the unfolded protein response, BiP dislocates from these ER stress sensors, enabling the activation of their downstream signaling pathways, resulting in p38 MAPK and JNK activation and the translocation of the X-box binding protein 1 (XBP1s) into the nucleus to initiate lipid biosynthesis [50,58]. These processes collectively lower protein synthesis and stabilize stressed cells but can also result in apoptosis. However, no reduction in the viability of Crenolanib-treated HSC was observed when ≤1 µM Crenolanib was applied in the present study.

**Figure 8:**
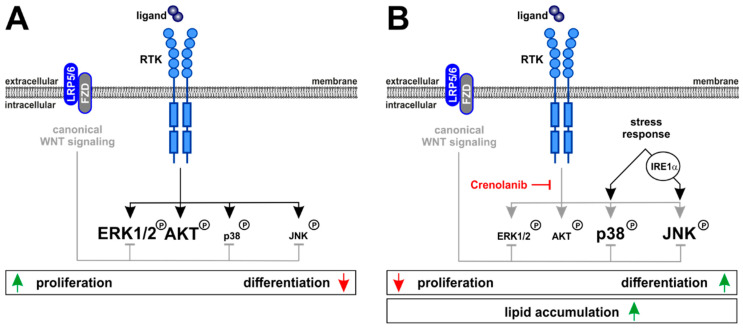
Schematic showing the molecular mechanisms that govern HSC behavior via Crenolanib. (**A**) The stimulation of RTK signaling using ligands, such as PDGF-BB, increases the phosphorylation of ERK1/2 and AKT, whereas p38 MAPK and JNK were only weakly phosphorylated after PDGF-BB exposure. (**B**) By contrast, blocking RTK signaling using Crenolanib led to p38 MAPK and JNK phosphorylation, resulting in reduced cell proliferation and the initiation of cell differentiation processes. Thus, RTK signaling was capable of balancing the opposing effects of cell proliferation and differentiation. In contrast, canonical WNT signaling suppressed proliferation and differentiation in HSC by inhibiting ERK1/2, p38 MAPK, and JNK activation, indicating that the regulatory functions of canonical WNT signaling are capable of sustaining quiescence in HSC. The accumulation of lipids, especially in response to high Crenolanib concentrations, suggested the activation of adaptive stress pathways and provided an explanation for p38 MAPK and JNK activation. IRE1α was found to be responsible for Crenolanib-mediated JNK phosphorylation and endodermal specification of HSC.

The initiation of developmental processes by inhibiting RTK-mediated cell proliferation prompted us to search for regulatory mechanisms that could preserve HSC characteristics. Canonical WNT signaling via β-catenin is a suitable option for maintaining quiescence in HSC, as demonstrated previously [59]. Blocking canonical WNT signaling through the application of the LRP5/6 inhibitor DKK1 enhanced various pathways involved in cell proliferation (ERK1/2) and differentiation (p38 MAPK, JNK), as demonstrated by this study. By contrast, the amplification of canonical WNT signaling via the GSK3β inhibitor TWS119 prevented MAPK activation. Thus, active canonical WNT signaling has regulatory functions that preserve quiescence in HSC. RTK signaling, in turn, promotes HSC proliferation but counteracts cell differentiation which is promoted by the phosphorylation of p38 MAPK and JNK. Quiescence, proliferation, and differentiation are opposing mechanisms that control the fate of HSC, and RTK signaling appears to be critically involved in balancing these processes (Figure 8).

## 5. Conclusions

This study identified Crenolanib as a promising agent that promotes the recovery of the liver from fibrosis. The beneficial effects of Crenolanib on the regression of fibrous scars in the chronically injured liver could be explained by the inhibition of RTK-mediated HSC proliferation. The induction of a stress response can result in the endodermal development of HSC or a reduction in protein synthesis through an adaptive mechanism associated with IRE1α activation, which might contribute to the improved recovery from fibrosis observed in Crenolanib-treated rats. However, before Crenolanib can be used to cure patients, the potential side effects of this substance must be carefully evaluated. The communication between multipotent pericytes (precursors of MSC) and endothelial cells involves PDGFRβ signaling and is essential for the prevention of microvascular dysfunction [60]. Stress induction through the interference of RTK signaling, as shown in the present study, or through the mediation of nutrition, as discussed elsewhere [50,58], can initiate developmental processes that will impair genuine MSC functions such as immune system suppression and tissue regeneration support.

## Data Availability

Not applicable.

## Supplementary Materials

The following are available online at https://www.mdpi.com/article/10.3390/cells10040804/s1, Table S1: Primer sets used for qPCR analysis. Table S2: Antibodies used for Western blot and immunofluorescence. Figure S1: Improved recovery of Crenolanib-treated rat livers from fibrosis. Figure S2: Crenolanib treatment lowered mesodermal but induced hepatocyte markers in isolated rat HSC. Figure S3: Crenolanib treatment of the HSC clone 5G4. Figure S4: GATA4 expression by HSC. Figure S5: Evaluation of p38 MAPK/MAPKAPK2 and JNK inhibitors in Crenolanib-treated HSC. Figure S6: Expression of dual-specificity phosphatases (Dusp/mitogen-activated protein kinase phosphatase/Mkp) in HSC after the short- and long-term treatment with Crenolanib. Figure S7: DUSP1 protein levels in HSC after Crenolanib treatment. Figure S8: Inhibition of FGF signaling in Crenolanib-treated HSC. Figure S9: IL1β and LPS-mediated p38 MAPK and JNK activation in HSC.

## Abbreviations

αSMA (α-smooth muscle actin); AFP (alpha fetoprotein); BiP (binding immunoglobulin protein/HSPA5); BSEP (bile salt export pump); CK18/19 (cytokeratin 18/19); COL (collagen); DAPI (4′,6-diamidino-2-phenylindole); DKK1 (dickkopf 1); DMSO (dimethyl sulfoxide); DUSP (dual-specificity phosphatase); EGF (epidermal growth factor); EPCAM (epithelial cell adhesion molecule); ER (endoplasmic reticulum); ERK1/2 (extracellular signal-regulated kinases 1/2); FCS (fetal calf serum); FGF (fibroblast growth factor); FGFR (fibroblast growth factor receptor); FLT3 (FMS-related tyrosine kinase 3); FOXA2/3 (forkhead box A2/3); GATA4 (GATA binding protein 4); GFAP (glial fibrillary acidic protein); GPBAR1/TGR5 (G protein-coupled bile acid receptor 1); GS (glutamine synthetase); GSK3b (Glycogen synthase kinase 3 beta); HE (hematoxylin/eosin); HGF (hepatocyte growth factor); HHEX (hematopoietically-expressed homeobox gene); HNF1α (hepatocyte nuclear factor 1α) HNF4α (hepatocyte nuclear factor 4α); HRP (horseradish peroxidase); HSC (hepatic stellate cells); IL1 (interleukin-1); IL1R (interleukin-1 receptor); IRAK4 (interleukin-1 receptor-associated kinase 4); IRE1α (inositol-requiring enzyme-1α); ITS (insulin-transferrin-selenite); JNK (c-Jun N-terminal kinases); LPS (lipopolysaccharide); MAPK (mitogen-activated protein kinase); MAPKAPK2 (mitogen-activated protein kinase-activated protein kinase 2); MRP2 (multidrug resistance-associated protein 2); MSC (mesenchymal stem cells); MKP (mitogen-activated protein kinase phosphatases); NTCP (sodium/taurocholate co-transporting polypeptide); PDGF (platelet-derived growth factor); PDGFR (platelet-derived growth factor receptor); RECA1 (rat endothelial cell antigen-1); RTK (receptor tyrosine kinase); SOX9 (sex determining region Y-box transcription factor 9); SOX17 (sex determining region Y-box 17); TAA (thioacetamide); TLR (toll-like receptor).

## References

[1] Kordes C., Sawitza I., Götze S., Häussinger D. (2013). Hepatic stellate cells support hematopoiesis and are liver-resident mesenchymal stem cells. Cell Physiol. Biochem..

[2] Kordes C., Sawitza I., Götze S., Schumacher E., Häussinger D. (2015). Beyond fibrosis: Stellate cells as liver stem cells. Zeitschrift Gastroenterologie.

[3] Kordes C., Sawitza I., Götze S., Herebian D., Häussinger D. (2014). Hepatic stellate cells contribute to progenitor cells and liver regeneration. J. Clin. Invest..

[4] Häussinger D., Kordes C. (2019). Space of Disse: A stem cell niche in the liver. Biol. Chem..

[5] da Silva Meirelles L., Chagastelles P.C., Nardi N.B. (2006). Mesenchymal stem cells reside in virtually all post-natal organs and tissues. J. Cell Sci..

[6] Friedenstein A.J., Chailakhjan R.K., Lalykina K.S. (1970). The development of fibroblast colonies in monolayer cultures of guinea-pig bone marrow and spleen cells. Cell Prolif..

[7] Farini A., Sitzia C., Erratico S., Meregalli M., Torrente Y. (2014). Clinical applications of mesenchymal stem cells in chronic diseases. Stem Cells Int..

[8] Pittenger M.F., Mackay A.M., Beck S.C., Jaiswal R.K., Douglas R., Mosca J.D., Moorman M.A., Simonetti D.W., Craig S., Marshak D.R. (1999). Multilineage potential of adult human mesenchymal stem cells. Science.

[9] Schwartz R.E., Reyes M., Koodie L., Jiang Y., Blackstad M., Lund T., Lenvik T., Johnson S., Hu W.-S., Verfaillie C.M. (2002). Multipotent adult progenitor cells from bone marrow differentiate into functional hepatocyte-like cells. J. Clin. Invest..

[10] Spitzhorn L.S., Kordes C., Megges M., Sawitza I., Götze S., Reichert D., Schulze-Matz P., Graffmann N., Bohndorf M., Wruck W. (2018). Transplanted human pluripotent stem cell-derived mesenchymal stem cells support liver regeneration in Gunn rats. Stem Cells Dev..

[11] Baertschiger R.M., Serre-Beinier V., Morel P., Bosco D., Peyrou M., Clement S., Sgroi A., Kaelin A., Buhler L.H., Gonelle-Gispert C. (2009). Fibrogenic potential of human multipotent mesenchymal stromal cells in injured liver. PLoS ONE.

[12] Marriott S., Baskir R.S., Gaskill C., Menon S., Carrier E.J., Williams J., Talati M., Helm K., Alford C.E., Kropski J.A. (2014). ABCG2pos lung mesenchymal stem cells are a novel pericyte subpopulation that contributes to fibrotic remodeling. Am. J. Physiol. Cell Physiol..

[13] Kramann R., Schneider R.K., DiRocco D.P., Machado F., Fleig S., Bondzie P.A., Henderson J.M., Ebert B.L., Humphreys B.D. (2015). Perivascular Gli1+ progenitors are key contributors to injury-induced organ fibrosis. Cell Stem Cell.

[14] Liu Y., Yang X., Jing Y., Zhang S., Zong C., Jiang J., Sun K., Lixin W., Gao L., Zhao X. (2015). Contribution and Mobilization of Mesenchymal Stem Cells in a mouse model of carbon tetrachloride-induced liver fibrosis. Sci. Rep..

[15] Ieronimakis N., Hays A., Prasad A., Janebodin K., Duffield J.S., Reyes M. (2016). PDGFRalpha signalling promotes fibrogenic responses in collagen-producing cells in Duchenne muscular dystrophy. J. Pathol..

[16] Trial J., Entman M.L., Cieslik K.A. (2016). Mesenchymal stem cell-derived inflammatory fibroblasts mediate interstitial fibrosis in the aging heart. J. Mol. Cell Cardiol..

[17] Kordes C., Häussinger D. (2013). Hepatic stem cell niches. J. Clin. Invest..

[18] Sawitza I., Kordes C., Reister S., Häussinger D. (2009). The niche of stellate cells within rat liver. Hepatology.

[19] Wake K. (1980). Perisinusoidal stellate cells (fat-storing cells, interstitial cells, lipocytes), their related structure in and around the liver sinusoids, and vitamin A-storing cells in extrahepatic organs. Int. Rev. Cytol..

[20] Wake K. (1971). “Sternzellen” in the liver: Perisinusoidal cells with special reference to storage of vitamin A. Am. J. Anat..

[21] Hendriks H.F., Verhoofstad W.A., Brouwer A., de Leeuw A.M., Knook D.L. (1985). Perisinusoidal fat-storing cells are the main vitamin A storage sites in rat liver. Exp. Cell Res..

[22] Friedman S.L., Roll F.J., Boyles J., Bissell D.M. (1985). Hepatic lipocytes: The principal collagen-producing cells of normal rat liver. Proc. Natl. Acad. Sci. USA.

[23] Bühring H., Battula V.L., Treml S., Schewe B., Kanz L., Vogel W. (2007). Novel Markers for the Prospective Isolation of Human MSC. Ann. N. Y. Acad. Sci..

[24] Claesson-Welsh L. (1994). Signal transduction by the PDGF receptors. Prog. Growth Factor Res..

[25] Houlihan D.D., Mabuchi Y., Morikawa S., Niibe K., Araki D., Suzuki S., Okano H., Matsuzaki Y. (2012). Isolation of mouse mesenchymal stem cells on the basis of expression of Sca-1 and PDGFR-α. Nat. Protoc..

[26] Morikawa S., Mabuchi Y., Kubota Y., Nagai Y., Niibe K., Hiratsu E., Suzuki S., Miyauchi-Hara C., Nagoshi N., Sunabori T. (2009). Prospective identification, isolation, and systemic transplantation of multipotent mesenchymal stem cells in murine bone marrow. J. Exp. Med..

[27] Artemenko Y., Gagnon A., Aubin D., Sorisky A. (2005). Anti-adipogenic effect of PDGF is reversed by PKC inhibition. J. Cell Physiol..

[28] Heldin C.H. (2013). Targeting the PDGF signaling pathway in tumor treatment. Cell Commun. Signal..

[29] Nazari M., Ni N.C., Lüdke A., Li S.-H., Guo J., Weisel R.D., Li R.-K. (2016). Mast cells promote proliferation and migration and inhibit differentiation of mesenchymal stem cells through PDGF. J. Mol. Cell. Cardiol..

[30] Qu B., Xia X., Wu H.H., Tu C.Q., Pan X.M. (2014). PDGF-regulated miRNA-138 inhibits the osteogenic differentiation of mesenchymal stem cells. Biochem. Biophys. Res. Commun..

[31] Breitkopf K., Roeyen C., Sawitza I., Wickert L., Floege J., Gressner A.M. (2005). Expression patterns of PDGF-A, -B, -C and -D and the PDGF-receptors alpha and beta in activated rat hepatic stellate cells (HSC). Cytokine.

[32] Kordes C., Brookmann S., Häussinger D., Klonowski-Stumpe H. (2005). Differential and synergistic effects of platelet-derived growth factor-BB and transforming growth factor-beta1 on activated pancreatic stellate cells. Pancreas.

[33] Zhang B.-B., Cai W.-M., Weng H.-L., Hu Z.-R., Lu J., Zheng M., Liu R.-H. (2003). Diagnostic value of platelet derived growth factor-BB, transforming growth factor-β1, matrix metalloproteinase-1, and tissue inhibitor of matrix metalloproteinase-1 in serum and peripheral blood mononuclear cells for hepatic fibrosis. World J. Gastroenterol..

[34] Zhou J., Deng Y., Yan L., Zhao H., Wang G. (2016). Serum platelet-derived growth factor BB levels: A potential biomarker for the assessment of liver fibrosis in patients with chronic hepatitis B. Int. J. Infect. Dis..

[35] Ikura Y., Morimoto H., Ogami M., Jomura H., Ikeoka N., Sakurai M. (1997). Expression of platelet-derived growth factor and its receptor in livers of patients with chronic liver disease. J. Gastroenterol..

[36] Wong L., Yamasaki G., Johnson R.J., Friedman S.L. (1994). Induction of beta-platelet-derived growth factor receptor in rat hepatic lipocytes during cellular activation in vivo and in culture. J. Clin. Invest..

[37] Galanis A., Ma H., Rajkhowa T., Ramachandran A., Small N., Cortes J., Levis M. (2014). Crenolanib is a potent inhibitor of FLT3 with activity against resistance-conferring point mutants. Blood.

[38] Lewis N.L., Lewis L.D., Eder J.P., Reddy N.J., Guo F., Pierce K.J., Olszanski A.J., Cohen R.B. (2009). Phase I study of the safety, tolerability, and pharmacokinetics of Oral CP-868,596, a highly specific platelet-derived growth factor receptor tyrosine kinase inhibitor in patients with advanced cancers. J. Clin. Oncol..

[39] Li X., Benjamin I.S., Alexander B. (2002). Reproducible production of thioacetamide-induced macronodular cirrhosis in the rat with no mortality. J. Hepatol..

[40] Koblihova E., Mrazova I., Vernerova Z., Ryska M. (2014). Acute liver failure induced by thioacetamide: Selection of optimal dosage in Wistar and Lewis rats. Physiol. Res..

[41] Laleman W., Elst I.V., Zeegers M., Servaes R., Libbrecht L., Roskams T., Fevery J., Nevens F. (2006). A stable model of cirrhotic portal hypertension in the rat: Thioacetamide revisited. Eur. J. Clin. Investig..

[42] Schindelin J., Arganda-Carreras I., Frise E., Kaynig V., Longair M., Pietzsch T., Preibisch S., Rueden C., Saalfeld S., Schmid B. (2012). Fiji: An open-source platform for biological-image analysis. Nat. Methods.

[43] Labun K., Montague T.G., Krause M., Cleuren Y.N.T., Tjeldnes H., Valen E. (2019). CHOPCHOP v3: Expanding the CRISPR web toolbox beyond genome editing. Nucleic Acids Res..

[44] Niles A.L., Moravec R.A., Hesselberth P.E., Scurria M.A., Daily W.J., Riss T.L. (2007). A homogeneous assay to measure live and dead cells in the same sample by detecting different protease markers. Anal. Biochem..

[45] Harding A., Cortez-Toledo E., Magner N.L., Beegle J.R., Coleal-Bergum D.P., Hao D., Wang A., Nolta J.A., Zhou P. (2017). Highly efficient differentiation of endothelial cells from pluripotent stem cells requires the MAPK and the PI3K pathways. STEM CELLS.

[46] Kempf H., Lecina M., Ting S., Zweigerdt R., Oh S. (2011). Distinct regulation of mitogen-activated protein kinase activities is coupled with enhanced cardiac differentiation of human embryonic stem cells. Stem Cell Res..

[47] Ventura J.J., Tenbaum S.P., Perdiguero E., Huth M., Guerra C., Barbacid M., Pasparakis M., Nebreda A.R. (2007). p38α MAP kinase is essential in lung stem and progenitor cell proliferation and differentiation. Nat. Genet..

[48] Arthur J.S., Ley S.C. (2013). Mitogen-activated protein kinases in innate immunity. Nat. Rev. Immunol..

[49] Dong C., Davis R.J., Flavell R.A. (2002). MAP kinases in the immune response. Annu Rev. Immunol..

[50] Hotamisligil G.S., Davis R.J. (2016). Cell signaling and stress responses. Cold Spring Harb. Perspect. Biol..

[51] Kanai-Azuma M., Kanai Y., Gad J.M., Tajima Y., Taya C., Kurohmaru M., Sanai Y., Yonekawa H., Yazaki K., Tam P.P.L. (2002). Depletion of definitive gut endoderm in Sox17-null mutant mice. Development.

[52] Barbera J.P.M., Clements M., Thomas P., Rodriguez T., Meloy D., Kioussis D., Beddington R.S. (2000). The homeobox gene Hex is required in definitive endodermal tissues for normal forebrain, liver and thyroid formation. Development.

[53] Tanaka T., Inazu T., Yamada K., Myint Z., Keng V.W., Inoue Y., Taniguchi N., Noguchi T. (1999). cDNA cloning and expression of rat homeobox gene, Hex, and functional characterization of the protein. Biochem. J..

[54] Watt A.J., Zhao R., Li J., Duncan S.A. (2007). Development of the mammalian liver and ventral pancreas is dependent on GATA4. BMC Dev. Biol..

[55] Delgado I., Carrasco M., Cano E., Carmona R., García-Carbonero R., Marín-Gómez L.M., Soria B., Martín F., Cano D.A., Muñoz-Chápuli R. (2014). GATA4 loss in the septum transversum mesenchyme promotes liver fibrosis in mice. Hepatology.

[56] Matsuda K., Kobune Y., Noda C., Ichihara A. (1994). Expression of GATA-binding transcription factors in rat hepatocytes. FEBS Lett..

[57] Rhyasen G.W., Starczynowski D.T. (2015). IRAK signalling in cancer. Br. J. Cancer.

[58] Salvado L., Palomer X., Barroso E., Vazquez-Carrera M. (2015). Targeting endoplasmic reticulum stress in insulin resistance. Trends Endocrinol. Metab..

[59] Kordes C., Sawitza I., Häussinger D. (2008). Canonical Wnt signaling maintains the quiescent stage of hepatic stellate cells. Biochem. Biophys. Res. Commun..

[60] Gerhardt H., Betsholtz C. (2003). Endothelial-pericyte interactions in angiogenesis. Cell Tissue Res..

