# Supplementary material for: Improved Recovery from Liver Fibrosis by Crenolanib
Source: Cells. 2021 Apr 4;10(4):804. doi: 10.3390/cells10040804 (PMC8067177; doi:10.3390/cells10040804)
Supplement: Supplementary file 1 [file cells-10-00804-s001.pdf]

cells

MDPI

Supplement

Improved recovery from liver fibrosis by Crenolanib

Doreen Reichert, Louisa Adolph, Jan Philipp Köhler, Tobias Buschmann, Tom Luedde, Dieter Häussinger, Claus Kordes*

Clinic of Gastroenterology, Hepatology and Infectious Diseases, Heinrich-Heine-University, Moorenstraße 5, 40225 Düsseldorf, Germany; *Correspondence: claus.kordes@hhu.de; Tel.: 0049-211-81-14445

Supplemental Table S1: Primer sets used for qPCR analysis.

Gene	Forward Primer	Reverse Primer	bp	Accession No.
**αSma**	GCACTACCATGTACCCAGGCA	TGCGTTCTGGAGGAGCAA	102	NM_031004.2
**Afp**	ACCTGACAGGGAAGATGGTG	GCAGTGGTTGATACCGGAGT	155	NM_012493.2
**albumin**	CTTCAAAGCCTGGGCAGTAG	GCACTGGCTTATCACAGCAA	221	NM_134326.2
**Bsep**	TACCAGGAAAAGCGTGTGTG	CCCAGTGATGACCCATAACC	197	NM_031760.1
**Ck18**	CAGAAGAACCGTGAGGAACTG	TTCATCGAGTCCAGGTCAATC	161	NM_053976.1
**Ck19**	CCTTCCGTGATTACAGCCAGT	CTGTCTCAAACTTGGTCCGGA	147	NM_199498.2
**cMet**	GCACCCCAAAGCTGGTAATA	GATCCGGTTGAACGATCACT	477	NM_031517.2
**Col1α2**	ACCTCAGGGTGTTCAAGGTG	CGGATTCCAATAGGACCAGA	222	NM_053356.1
**desmin**	AGCCTGGGTCAGAGACAGAA	TATCTCCTGCTCCCACATCC	155	NM_022531.1
**Dusp1**	GAGCTGTGCAGCAAACAGTC	CCAGGTACAGGAAGGACAGG	151	NM_053769.3
**Dusp2**	TCACAGCGGTTCTCAATGTC	GCCCCCACTATTCTTCACTG	158	NM_001012089.1
**Dusp4**	CATGGAAGCCATCGAATACA	AACTCGAAAGCCTCCTCCA	150	NM_022199.1
**Dusp5**	ACAAGTGGATCCCTGTGGAG	TGAGGTAAGCCATGCAGATG	162	NM_133578.1
**Dusp6**	GCTGCTGCTCAAGAAACTCA	CAGACTCAATGTCCGAGGAAG	200	NM_053883.2
**Dusp7**	CACTGGAGCCAGAACCTCTC	CATCTTCTGCATCAGGTAGGC	150	NM_001100547.1
**Dusp8**	CATCTGTGAGAGCCGTTTCA	AGAAATGCCAGCCAGACAGT	148	NM_001108510.1
**Dusp9**	GAAGCTGAAGAGTGGGATGC	CCACTGAAGCTGGTTTCACA	146	NM_001037973.1
**Dusp10**	AGCAGGATGCTCAGGACCTA	GCAAGTTCTGCTTGTTGCTG	150	NM_001105734.1
**Dusp16**	CAGCGAGATGTCCTCAACAA	AGGGCAGGATTTTCTCACAA	154	NM_001106624.2
**Egf**	CTGTGATTGAAATGGCCGATCT	CCTGTTTTGACCAGTCCTCTTG	164	NM_012842.1
**Epcam**	TGCATACTGCACTTCAGGACA	GGAACAAGGACTCCCCCTTTA	195	NM_138541.1
**Fgf1**	GGCCACTTCTTGAGGATTCTTC	GTATAAAAGCCCTTCGGTGTCC	165	NM_012846.1
**Fgf2**	GAACCGGTACCTGGCTATGA	CCGTTTTGGATCCGAGTTTA	182	NM_019305.2
**Fgf7**	CTGTGGCAGTTGGAATTGTGG	CGCTGTGTGTCCATTTAGCTG	174	NM_022182.1
**Fgf9**	GGACTCTACCTCGGCATGAA	GTATCTCCTTCCGGTGTCCA	150	NM_012952.1
**Fgf10**	GTGGAAATCGGAGTTGTTGCC	CCGTTGTGCTGCCAGTTAAAA	173	NM_012951.1
**Fgf12**	CGGGGTGTTCAGCAAAGT	AGTCGCTGTTTTCGTCCTTG	170	NM_13814.1
**Fgfr1**	AGCTGGCGTCTCTGAATATG	GGTTGGGTTTGTCCTTATCC	149	NM_024146.1
**Fgfr2**	CGAATACGCATCGAAAGGCAA	GCTGCCAAGTCTCGATGGATA	195	NM_001109896.1
**Fgfr3**	GAGGATGCTGGGGTCTACAG	CGGTCGAGTCCAGTAAGGAG	153	NM_053429.1
**Fgfr4**	GCTATCTGCTGGATGTGCTG	CTGCCGTTGATAACGATGTG	169	NM_001109904.1
**Foxa2**	GTGAAGATGGAAGGGCACGAG	TGACATGTTCATGGAGCCTGC	186	NM_012743.1
**Gpbar1/Tgr5**	TTCTCTCTGTCCGAGTGTTGG	CACAGCAAAAAGAGCAGTGTG	160	NM_177936.1
**Gs**	ACGCTGCAAGACCCGTACTC	TGGAGCCTTCAGACTGAAACG	101	NM_017073.4
**Hgf**	CGAGCTATCGCGGTAAAGAC	TGTAGCTTTCACCGTTGCAG	165	NM_017017.2
**Hnf1α**	CAGCCACAACCATTCACATC	CGTTGGAGTCAGAACTCTGGT	132	NM_001306179.2
**Hnf4α**	AAATGTGCAGGTGTTGACCA	CACGCTCCTCCTGAAGAATC	178	NM_022180.2
**Hprt1**	AAGTGTTGGATACAGGCCAGA	GGCTTTGTACTTGGCTTTTCC	145	NM_012583.2
**Mrp2**	TCATCCCTCACAAACTGCCTC	ATTCATCCTCAGACTCCCCGA	162	NM_012833.2
**Ntcp**	AACATTGAAGCTCTGGCCATC	CACTGAAGCTGGAGCAGGTG	130	NM_017047.2
**Rps6**	GGAAGCGCAAGTCTGTCCGA	AGGTCCCAACCGACGAGGCA	131	NM_017160.1
**Sox9**	TCTCTCCTAACGCCATCTTCA	AGATCAACTTTCCCAGCTTGC	163	NM_080403.2

cells

MDPI

Supplemental Table S2: Antibodies used for western blot and immunofluorescence.

Antibody	Species	Company	Order No.
**αSMA**	mouse	Dako	00072869
**γ-tubulin**	mouse	Sigma-Aldrich	T5326
**AKT**	rabbit	Cell Signaling	9272
**BiP**	rabbit	Cell Signaling	3183
**CD90**	rabbit	Cell Signaling	13801
**CD44**	mouse	Cell Signaling	5640
**CD146**	mouse	Life Span Biosciences	LS-C35841
**collagen 4**	rabbit	Abcam	ab6586
**CK18**	mouse	Acris	BM2275P
**CK19**	rabbit	Novus Bio	NB100-687
**CK19**	mouse	Progen	65129
**desmin**	mouse	Dako	00073088
**desmin**	rabbit	Cell Signaling	5332S
**DUSP1 / MPP-1**	rabbit	Merck Millipore	07-535
**FOXA2**	rabbit	Cell Signaling	8186
**GATA4**	mouse	Santa Cruz Biotech.	sc-25310
**GFAP**	mouse	Chemicon	mab3402
**HHEX**	mouse	Chemicon	mab10071
**HNF4α**	rabbit	Cell Signaling	3113
**IRAK4**	rabbit	Cell Signaling	4363
**IRE1α**	rabbit	Santa Cruz Biotech	sc-390960
**nestin**	mouse	Santa Cruz Biotech.	sc-33677
**PDGFR-α**	rabbit	Cell Signaling	3164
**PDGFR-β**	rabbit	Cell Signaling	3169
**phospho-p38 MAPK (Thr180/Tyr182)**	rabbit	Cell Signaling	9211
**phospho AKT (Ser473)**	rabbit	Cell Signaling	4058
**phospho-ERK1/2 (Thr202/Tyr204)**	mouse	Cell Signaling	9106
**phospho-JNK (Thr183/Tyr185)**	rabbit	Cell Signaling	4671
**phospho-MAPKAPK2 (Thr222)**	rabbit	Celll Signaling	3316
**SOX17**	goat	R&D Systems	AF1924
**SPARCL1**	mouse	Santa Cruz Biotech.	sc-514275
**total ERK1/2**	rabbit	Millipore	06-182
**total JNK**	mouse	BD	554285
**total p38 MAPK**	mouse	Cell Signaling	9212
**total MAPKAPK2**	rabbit	Cell Signaling	3042
**vimentin**	mouse	Dako	20022872
**anti-ms-IgG, Cy3**	donkey	Millipore	AP192C
**anti-ms-IgG, FITC**	donkey	Millipore	AP192F
**anti-ms-IgG, HRP**	donkey	Millipore	AP192P
**anti-rb-IgG, Cy3**	donkey	Millipore	AP182C
**anti-rb-IgG, FITC**	donkey	Millipore	AP182F
**anti-rb-IgG, HRP**	donkey	Millipore	AP182P

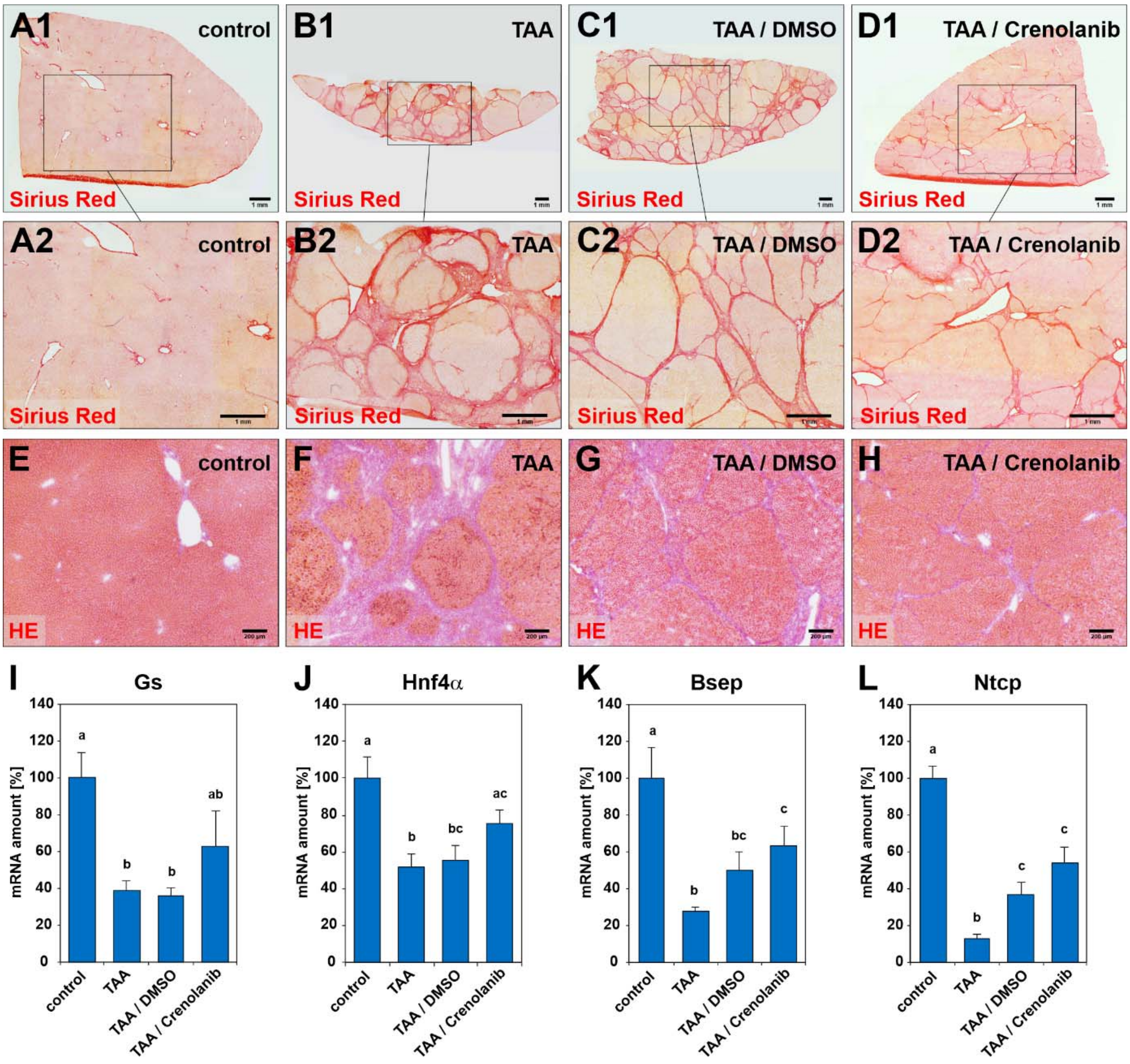

Supplemental Figure S1: Improved recovery of Crenolanib-treated rat livers from fibrosis. (A1-D2) Liver sections from rats were stained with Sirius Red to show connective tissue deposits. A2-D2 (scale bars: 200 µm) show magnifications of A1-D1 (scale bars: 1 mm). Complete tissue sections were scanned at low magnification (4x objective) and images were composed by the CellSens Dimension imaging software (Olympus). (E-H) HE colorings of liver tissue sections from rats (scale bars: 200 µm). (A1, A2, E) Rats were left untreated or (B1, B2, F) treated with TAA for 18 weeks to induce liver fibrosis. The livers could recover from liver fibrosis for 14 days after cessation of TAA treatment in the (C1, C2, G) absence and (D1, D2, H) presence of Crenolanib. (C1, C2, G) The control group with fibrosis, allowed to recover for 14 days, was treated with the vehicle DMSO used as a solvent of Crenolanib. Stronger regression of fibrotic scars was observed in the (D1, D2, H) Crenolanib-treated rats compared to the (C1, C2, G) DMSO-treated animals. (I-L) Whole liver samples of the rats were investigated by qPCR with respect to the expression of hepatocyte-specific genes. The expression of (I) Gs, (J) Hnf4α, (K) Bsep, and (L) Ntcp tended to increase in the liver of the Crenolanib-treated rats in comparison to the DMSO control animals. This further suggested that Crenolanib supported the recovery of the rats from TAA-induced liver fibrosis ($n = 4$-6; $p < 0.05$; significant differences are indicated by different letters).

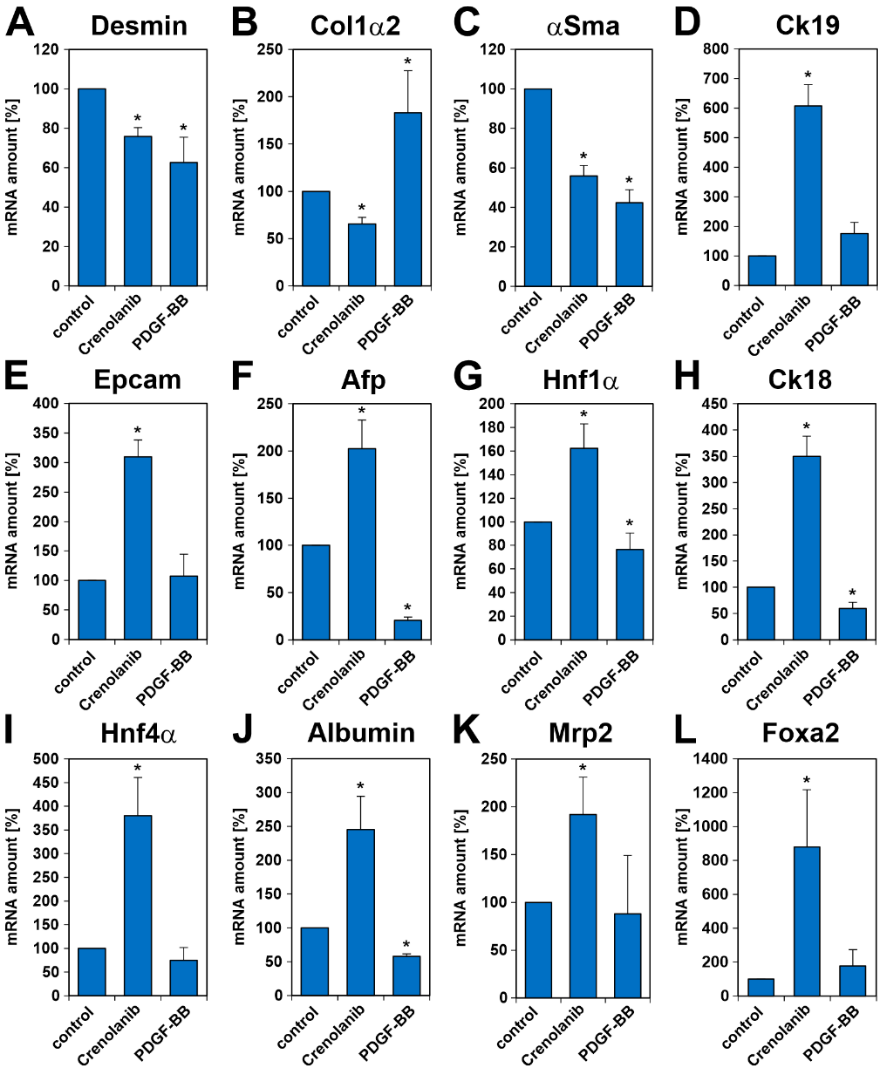

Supplemental Figure S2: Crenolanib treatment lowered mesodermal but induced hepatocyte markers in isolated rat HSC. (A-L) Primary HSC cultures (n = 5-40) were treated with Crenolanib (0.1 µM) or 20 ng/ml PDGF-BB in serum-free medium for 7 days and finally analyzed by qPCR. (A-C) The mRNA of genes typically expressed by mesenchymal cells, (D-F) liver progenitor cells and (G-L) hepatocytes were measured by qPCR in comparison to untreated HSC (control, 100%). While the expression of mesodermal genes decreased after Crenolanib treatment, the mRNA levels of liver progenitor cell- and hepatocyte-associated genes increased significantly. In contrast, PDGF-BB treatment suppressed or had no effect on the expression of hepatocyte markers in HSC. Only the expression of Col1α2 increased in HSC in the presence of PDGF-BB (*$p<0.05$ in comparison to control HSC).

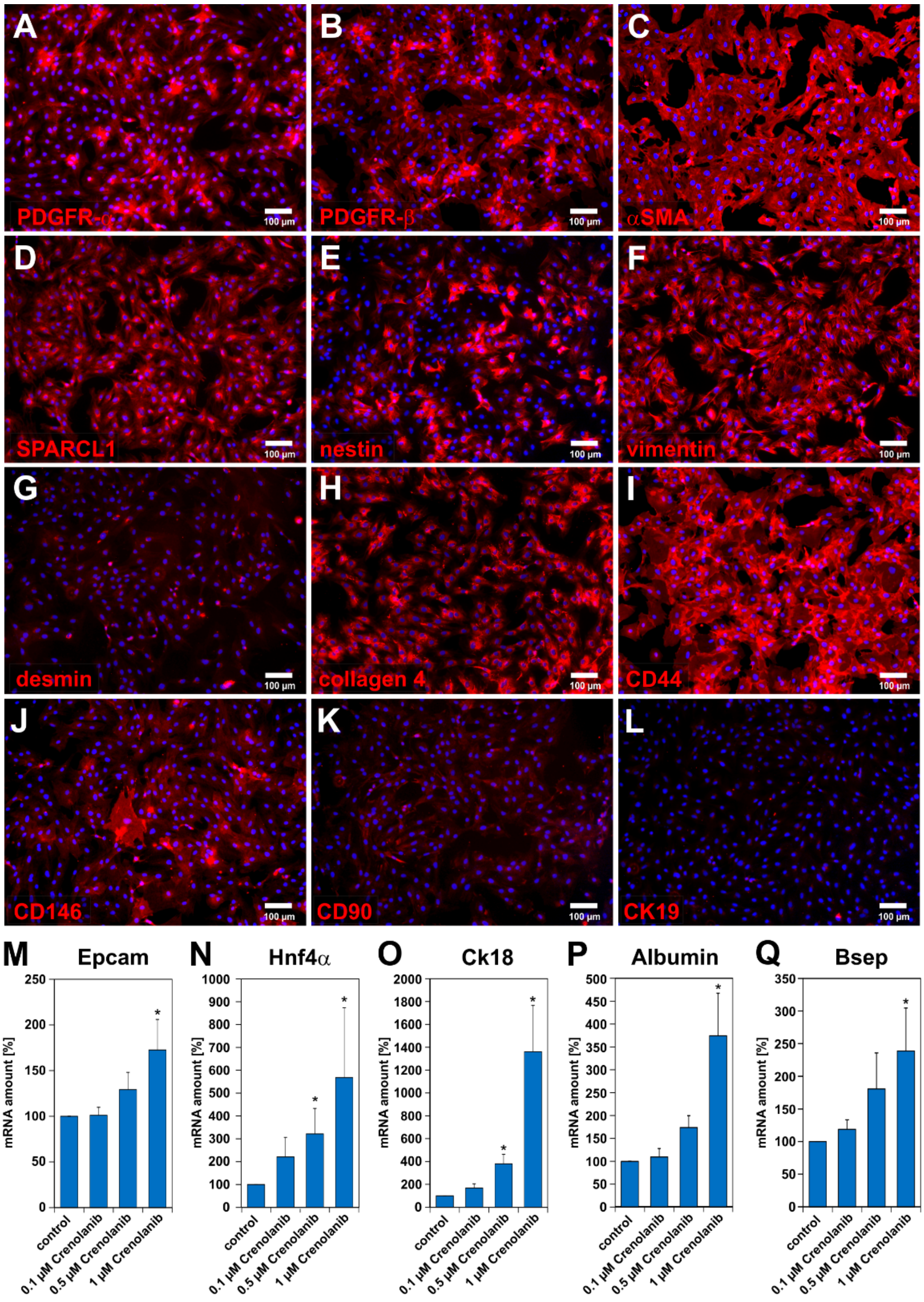

Supplemental Figure S3: Crenolanib treatment of the HSC clone 5G4. (A-K) Clonally expanded rat HSC of the clone 5G4 were characterized by immunofluorescence with markers for stellate cells and other MSC. PDGF receptors and typically stellate cell markers (αSMA, SPARCL1, nestin, vimentin, desmin, collagen 4) were found. (I, J) Also MSC markers such as CD44 and CD146 were expressed by the HSC clone 5G4. (K) In contrast to this, CD90 was only weakly expressed and (L) the epithelial cell marker CK19 was absent (scale bars in A-L: 100 µm). (M-Q) The treatment of the HSC clone 5G4 with various concentrations of Crenolanib upregulated the expression of liver progenitor cell- (*Epcam*) and hepatocyte-associated genes as investigated by qPCR (n = 3; *$p<0.05$).

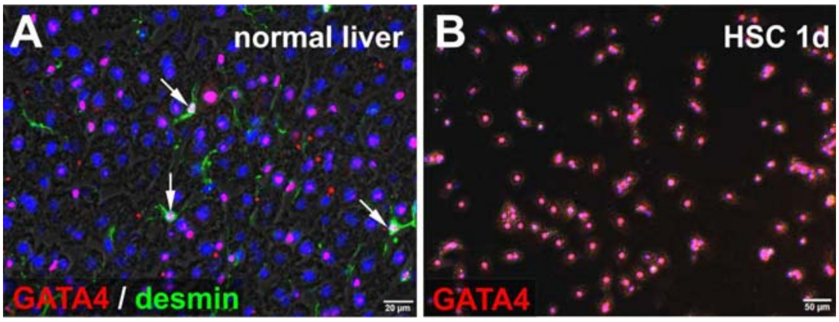

Supplemental Figure S4: GATA4 expression by HSC. (A) Immunofluorescence of GATA4 in tissue sections of normal rat liver. Co-staining of desmin (green) and GATA4 (red) indicated that GATA4 was already detectable in HSC *in situ* (arrows). In addition to HSC, GATA4 was also expressed to various degrees by hepatocytes. (B) Strong nuclear localization of GATA4 was maintained in freshly isolated HSC cultured for 1 day (scale bars in A and B: 50 µm).

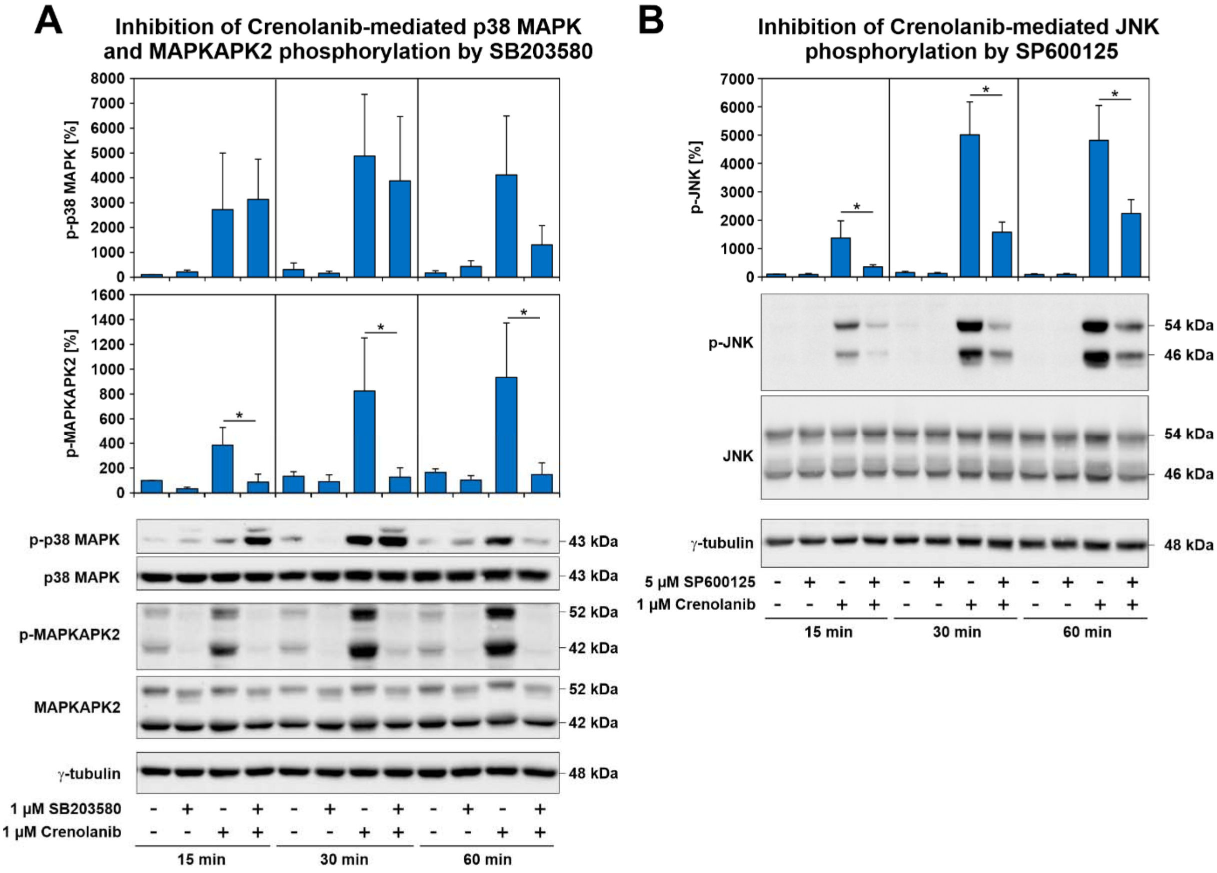

Supplemental Figure S5: Evaluation of p38 MAPK/MAPKAP2 and JNK inhibitors in Crenolanib-treated HSC. (A) The suitability of 1 µM SB203580 to suppress p38 MAPK signaling was tested by Western blot using antibodies against p38 MAPK and its downstream signaling element MAPKAPK2 in its phosphorylated form. Densitometry analysis of protein bands revealed that Crenolanib-mediated p38 MAPK phosphorylation was not significantly altered by SB203580, but MAPKAPK2 phosphorylation was significantly prevented by this inhibitor, indicating successful inhibition of p38 MAPK signaling by SB203580 in HSC (n = 3; *p < 0.05). (B) The suitability of the JNK inhibitor SP600125 (5 µM) to diminish Crenolanib-induced JNK phosphorylation in HSC was also tested by Western blot and was found to be able to reduce phosphorylated JNK significantly (n = 3; *p < 0.05). The protein γ-tubulin as well as total p38 MAPK and total JNK served as a loading controls and were used for normalization.

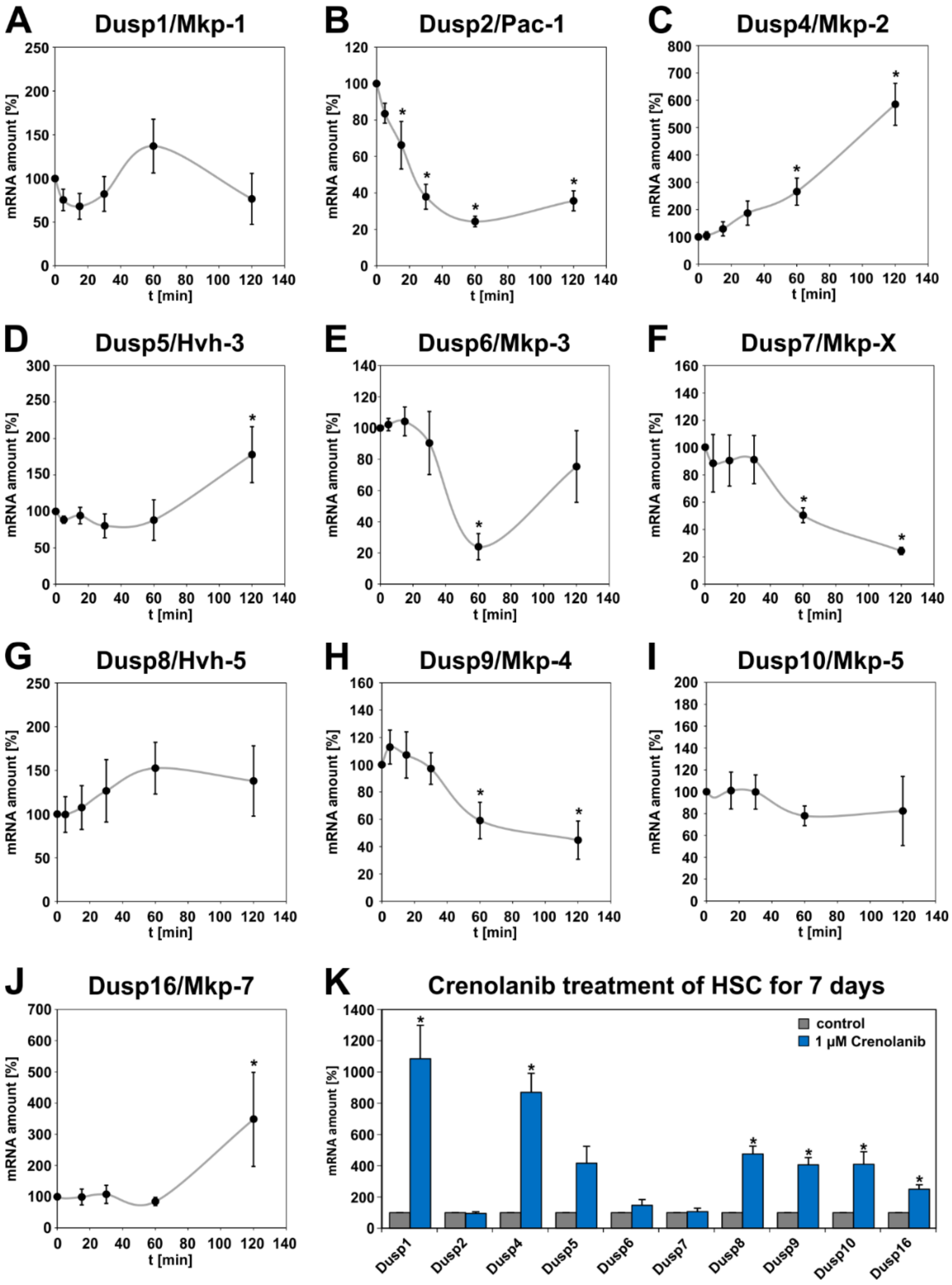

Supplemental Figure S6: Expression of dual-specificity phosphatases (Dusp/mitogen-activated protein kinase phosphatase/Mkp) in HSC after short- and long-term treatment with Crenolanib. (A-J) HSC were treated with 1 µM Crenolanib and analyzed with respect to *Dusp* expression by qPCR at indicated time points (n = 4; *$p < 0.05$). Short-term stimulation of HSC with Crenolanib for up to 120 min indicated that some *Dusp* might be involved in elevated p38 MAPK and JNK signaling as indicated by downregulation of mRNA amounts. (K) However, analysis of long-term stimulated HSC over 7 days by qPCR revealed either no alteration or an increase in *Dusp* expression, which could not explain the sustained kinase activation in response to Crenolanib (n = 3; *$p < 0.05$).

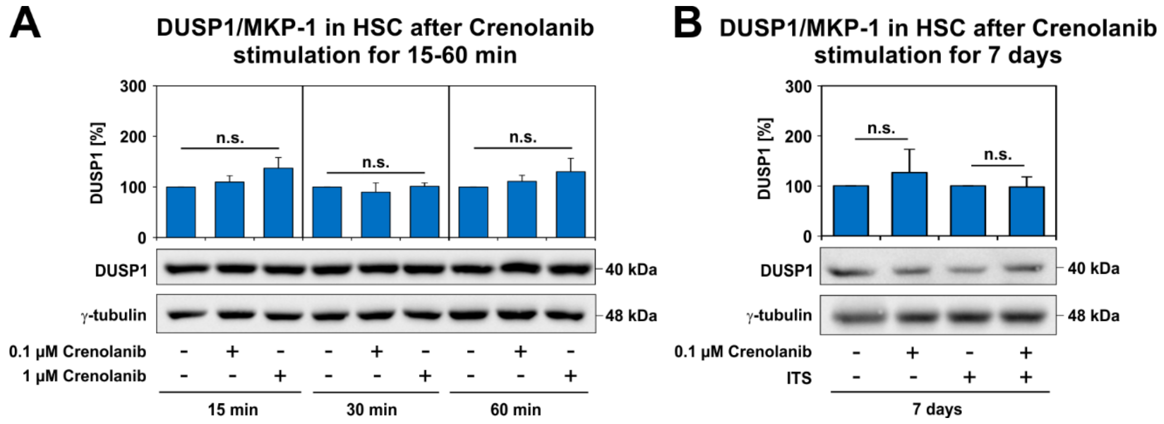

Supplemental Figure S7: DUSP1 protein levels in HSC after Crenolanib treatment. Since *Dusp1* expression was found to be highly upregulated in HSC by Crenolanib, this phosphatase was also analyzed at protein level by Western blot. (A) DUSP1 protein levels were analyzed in HSC cultured in medium without ITS at indicated time points (n = 3). (B) The presence of ITS in the culture medium had no obvious effect on the DUSP1 levels after treatment of HSC with 0.1 µM Crenolanib for 7 days (n = 3; $p < 0.05$).

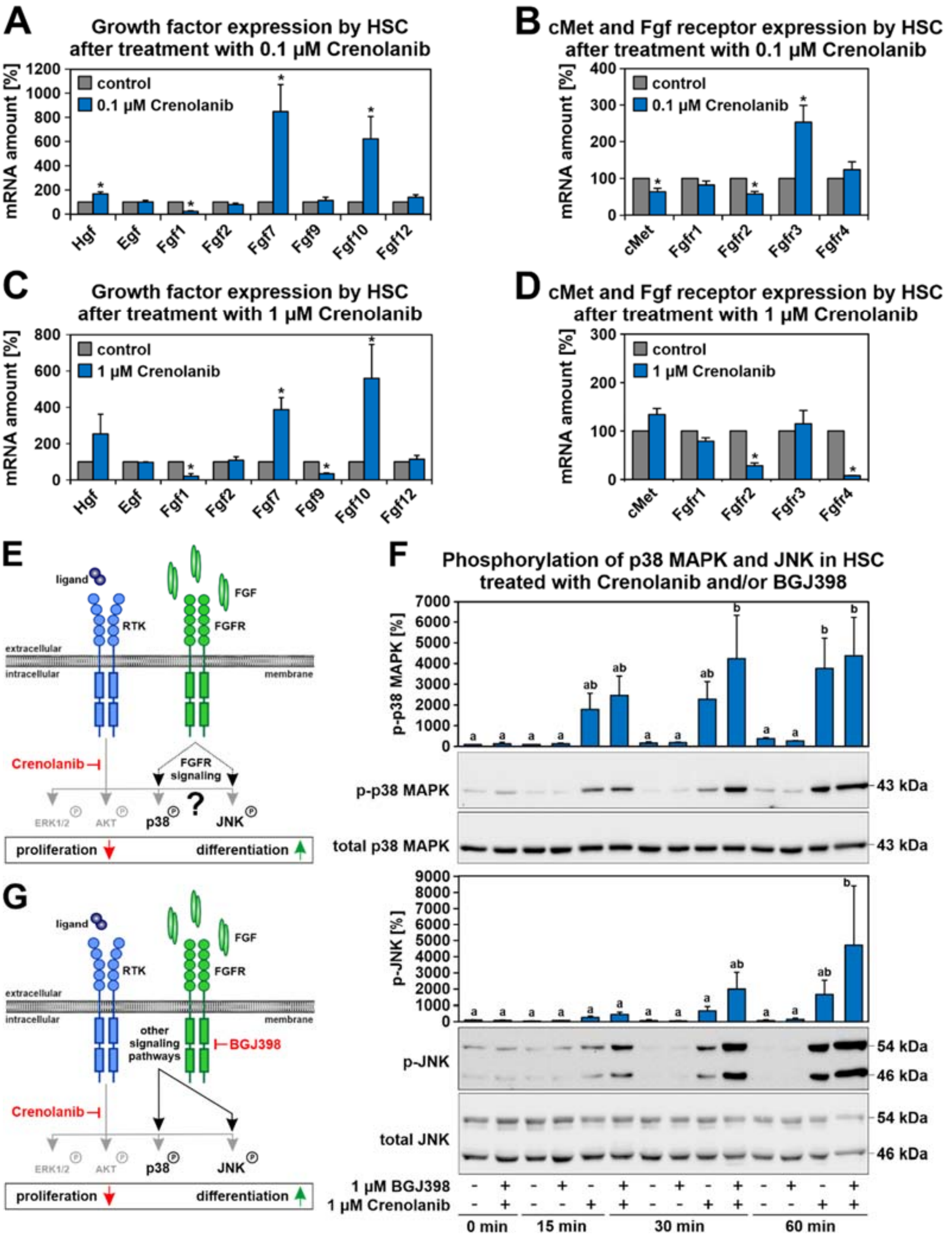

Supplemental Figure S8: Inhibition of FGF signaling in Crenolanib-treated HSC. To identify the mechanism responsible for Crenolanib-mediated endodermal specification of HSC, (A, C) growth factors and (B, D) receptors known to control developmental fate decisions in stem/progenitor cells were analyzed by pPCR in HSC treated with 0.1 or 1 µM Crenolanib for 7 days (n = 4-10; *$p < 0.05$). Hepatocyte growth factor (*Hgf*), *Fgf7* and *Fgf10* were found to be upregulated in HSC in response to Crenolanib treatment, while only the mRNA levels of *Fgfr3* increased significantly at 0.1 µM Crenolanib. Elevating the Crenolanib amount to 1 µM prevented increased Fgfr3 expression. (E) To evaluate the hypothesis that autocrine or paracrine stimulation of HSC by growth factors is involved in Crenolanib-mediated endodermal specification of HSC, the FGFR inhibitor BGJ398 was used. (F) HSC were pre-treated with 1 µM BGJ398 before 1 µM Crenolanib was applied. Western blot analysis indicated no inhibition of p38 MAPK and JNK phosphorylation by BGJ398 after short-term stimulation with Crenolanib (15-60 min; n = 3; $p < 0.05$; significant differences are indicated by different letters). (G) This suggested that FGFR signaling had little or no effect on Crenolanib-mediated p38 MAPK and JNK activation. Other signaling pathways seem to be responsible for this process.

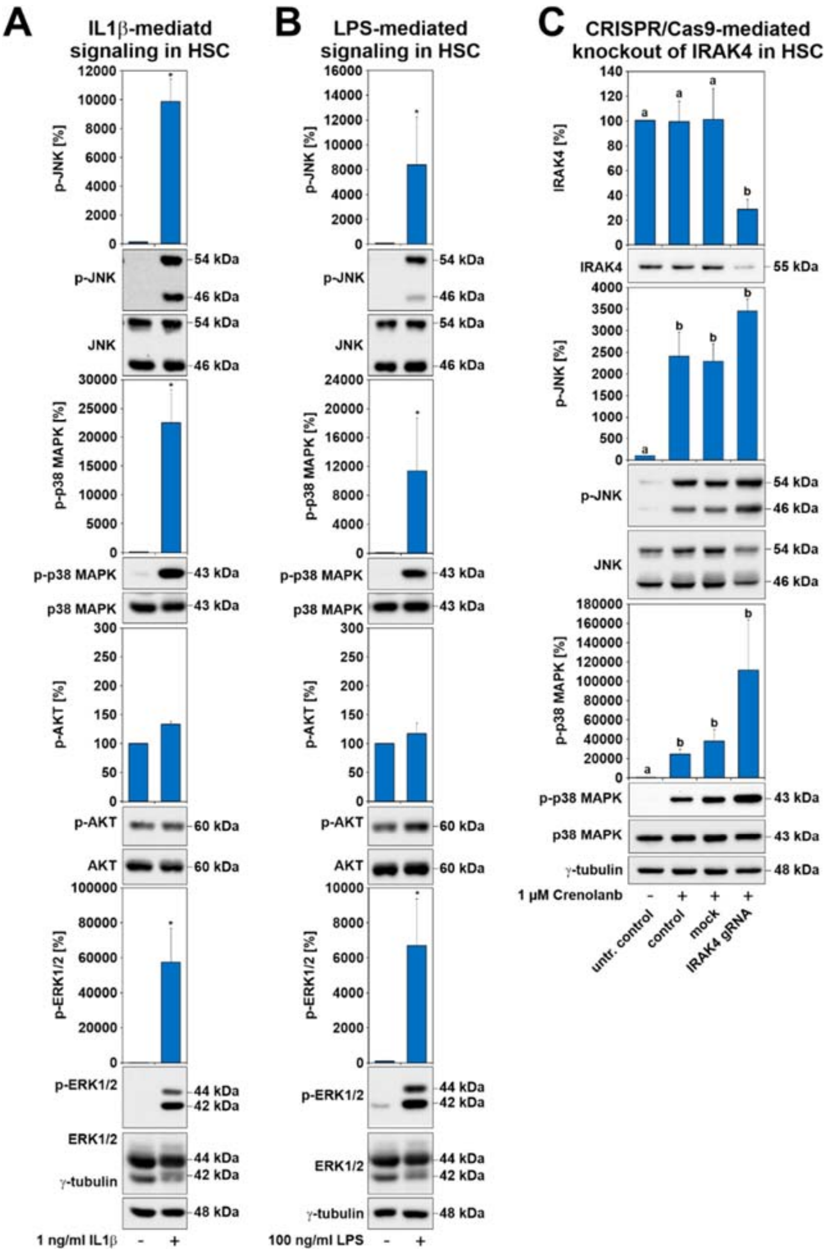

Supplemental Figure S9: IL1β and LPS-mediated p38 MAPK and JNK activation in HSC. IL1β and LPS are known to trigger p38 MAPK and JNK activation via IL1R and TLR4 signaling. Therefore, activated HSC were treated with 1 ng/ml IL1β or 100 ng/ml LPS, to evaluate the concept that Crenolanib might exert its effects on p38 MAPK and JNK signaling through these receptors. (A, B) IL1β and LPS were able to activate p38 MAPK and JNK in HSC but increased also ERK1/2 phosphorylation. In contrast, AKT phosphorylation remained unchanged in HSC after stimulation with IL1β and LPS (n = 3; *$p < 0.05$). (C) IRAK4 is a downstream factor of IL1R and TLR4 pathways. Therefore, the IRAK4 gene was deleted by CRISPR/Cas9-mediated knockout in HSC. IRAK4 protein levels were significantly reduced by this method, but HSC treated with 1 µM Crenolanib still showed p38 MAPK and JNK phosphorylation compared to the mock control transfected with Cas9 only (n = 3; $p < 0.05$; significant differences are indicated by different letters).
